# Investigation of the mechanism by which miR-223-3p inhibits reflux esophagitis through targeting the NLRP3 inflammasome

**DOI:** 10.1186/s12876-025-03836-9

**Published:** 2025-05-13

**Authors:** Shuying Lin, Binbin Zheng, Ruchen Wu, Qiuli Wu, Xiangbo Chen

**Affiliations:** 1 Endoscopy Room, Quanzhou First Hospital Affiliated to Fujian Medical University, 248 - 252 East Street, Licheng District, Quanzhou, Fujian Province 362000 China; 2 Department of Clinical Medicine, Xuzhou Medical University, Yunlong District, Xuzhou, Jiangsu Province, China

**Keywords:** Reflux esophagitis, NLRP3 inflammasome, MiR- 223 - 3p, Apoptosis

## Abstract

**Background:**

Reflux esophagitis is a common gastrointestinal disorder characterized by significant inflammatory responses. The NLRP3 inflammasome plays a crucial role in inflammation, and miR- 223 - 3p has been found to inhibit its expression by targeting NLRP3 mRNA. This study aims to further investigate the mechanism by which miR- 223 - 3p inhibits reflux esophagitis through targeting the NLRP3 inflammasome.

**Methods:**

A reflux esophagitis cell model was constructed to assess the expression levels of miR- 223 - 3p and NLRP3. Overexpression and inhibition techniques were used to study the effects of miR- 223 - 3p on the NLRP3 inflammasome. qPCR and Western blot analyses were employed to detect the expression of related inflammatory factors, and flow cytometry was used to assess cell apoptosis and cell cycle changes.

**Results:**

The study found that miR- 223 - 3p was significantly downregulated in the reflux esophagitis model, while NLRP3 and its downstream inflammatory factors were significantly upregulated. Overexpression of miR- 223 - 3p markedly inhibited NLRP3 expression, reduced the release of inflammatory factors, decreased cell apoptosis, promoted cell cycle progression, and enhanced cell viability. Overexpression of NLRP3 reversed these protective effects of miR- 223 - 3p, further confirming that miR- 223 - 3p alleviates inflammation by inhibiting the activation of the NLRP3 inflammasome.

**Conclusion:**

This study demonstrates that miR- 223 - 3p plays a key role in reducing inflammation and cellular damage in reflux esophagitis by targeting the NLRP3 inflammasome. These findings provide new insights and potential therapeutic targets for the treatment of reflux esophagitis.

**Supplementary Information:**

The online version contains supplementary material available at 10.1186/s12876-025-03836-9.

## Introduction

Gastroesophageal reflux disease (GERD) is defined as a condition where the reflux of stomach contents into the esophagus causes troublesome symptoms and complications [1]. Common triggers include stress, overeating, high-fat diets, and alcohol consumption, with symptoms such as bloating, indigestion, chest pain, burning sensation, and heartburn [2]. Nighttime heartburn, in particular, can lead to sleep disturbances, affecting daytime quality of life [3]. GERD is widely recognized as a major cause of esophagitis due to the reflux of gastric acids, bile salts, and other harmful substances, which can trigger reflux esophagitis [4]. Complications of reflux esophagitis include esophageal ulcers, strictures, and the development of Barrett's esophagus, a precursor to esophageal adenocarcinoma [5]. Reflux esophagitis damages the normal squamous epithelium of the esophagus, promoting intestinal metaplasia of Barrett's esophagus. Studies have demonstrated that both GERD and Barrett's esophagus are associated with chronic enrichment of pro-inflammatory cytokines, including IL- 1β, IL- 6, IL- 8, and TNF-α [6]. GERD enhances esophageal epithelial permeability to dietary allergens and promotes inflammatory cascades [7, 8]. The inflammatory injury results in the replacement of the distal esophageal squamous epithelium by cardiac mucosa with intestinal metaplasia, a pathological transformation clinically defined as Barrett's esophagus [9]. To sum up, understanding the pathogenesis of reflux esophagitis and developing effective treatment strategies are crucial to prevent the progression to Barrett's esophagus and esophageal adenocarcinoma.

One of the main pathways leading to inflammatory diseases is the activation of inflammasomes. The NLRP3 (NOD-, LRR-, and pyrin domain-containing protein 3) inflammasome is one of the most extensively studied inflammasome complexes and can be activated by various stimuli [10]. Over the past decade, the activation and regulatory mechanisms of the NLRP3 inflammasome have been a focus of research [11]. Martinon et al. first described the inflammasome as a large multiprotein complex required for caspase- 1 processing and activation of the inflammatory cytokine interleukin- 1β (IL- 1β) [12]. The NLRP3 inflammasome consists of the sensor protein NLRP3, the adaptor protein ASC, and the effector protein caspase- 1. Its activation is thought to be a two-step process requiring a priming signal and an NLRP3 activation signal. The priming step triggers NF-κB-dependent upregulation of NLRP3 and IL- 1β expression and lowers the activation threshold of NLRP3 through additional post-translational modifications [13, 14]. The second step involves the recognition of NLRP3 activators, inducing NLRP3 activation and inflammasome formation.

In recent years, the post-transcriptional regulatory role of miRNAs on inflammasomes has gained significant attention. miRNAs can inhibit or enhance the gene expression of inflammasomes by binding to their mRNA targets [15]. MicroRNAs (miRNAs) are small non-coding RNAs that regulate gene expression by promoting the degradation of target mRNAs or inhibiting their translation [16]. miRNAs play critical roles in various cellular functions and events such as cell proliferation, metabolism, and tumorigenesis [17]. While many miRNAs are involved in the progression of esophageal adenocarcinoma [18, 19], research on key miRNAs in reflux esophagitis is relatively scarce. Recent studies have shown that miR- 223 - 3p is significantly upregulated in reflux esophagitis tissues compared to controls [20]. In 2012, Bauernfeind et al. identified miR- 223 - 3p as the first miRNA directly regulating NLRP3. miR- 223 - 3p is highly expressed in myeloid cells, particularly in neutrophils, and lowly expressed in B cells and T cells, which is opposite to the expression pattern of NLRP3 [21]. Moreover, miR- 223 - 3p expression decreases during monocyte differentiation, while NLRP3 increases, suggesting a role for miR- 223 - 3p in this process [22]. NLRP3 displays a conserved binding site for miR- 223 - 3p in its 3'UTR region, and binding of miR- 223 - 3p results in reduced NLRP3 activity. In vitro studies have shown that mutations in the NLRP3 target region can completely abolish miR- 223-mediated regulation of NLRP3 3'UTR [21]. However, studies on miR- 223 - 3p levels in reflux esophagitis remain limited.

The regulatory role of miR- 223 - 3p on NLRP3 in various physiological and pathological processes has been widely reported. For example, miR- 223 - 3p targets NLRP3 to regulate cartilage degeneration [22]; in breast cancer, miR- 223 - 3p induces NLRP3 inflammasome inactivation, inhibits tumor growth, and enhances antitumor immunity [23]; miR- 223 - 3p also regulates NLRP3 expression in immune cells [24]. There is evidence that miR- 223 - 3p-mediated activation of the NLRP3 inflammasome and pyroptosis are involved in the development of colitis [25]; targeting NLRP3 inhibits Staphylococcus aureus-induced inflammasome activation and pyroptosis [26]. Furthermore, studies have reported the activation of the NLRP3 inflammasome in Barrett's esophagus cells [27], and activation of the NLRP3 inflammasome has also been observed in esophageal epithelial cells stimulated by acidic bile salts [28]. While most studies show that high expression of miR- 223 - 3p inhibits NLRP3 inflammasome activation, a recent study indicated that in a gastric acid reflux esophagitis rat model, miR- 223 - 3p expression was significantly higher in reflux esophagitis tissues compared to controls, and its expression gradually decreased as reflux esophagitis progressed from the acute to the chronic stage [29]. This contradictory phenomenon requires further research to clarify the regulatory role of miR- 223 - 3p on the NLRP3 inflammasome in reflux esophagitis.

Therefore, we aimed to investigate the role of miR- 223 - 3p on NLRP3 inflammasome in the pathogenesis of GERD. We first established the acid and bile salts-induced injury model of HET- 1 A cells to mimic GERD. In miR- 233 - 3p overexpression and knockdown cell lines with or without NLRP3 overexpression, we then investigate the function of miR- 223 - 3p-mediated NLRP3 inflammasome expression in reflux esophagitis.

## Methods

### Reagents

Fetal bovine Serum (FSD500)(Excell Bio,China); Penicillin streptomycin solution (100X)(C0222)(Beyotime,China); Human Esophageal epithelial cell Complete medium (CM-H031) (Pricella,China); Cell/Tissue Total RNA Isolation Kit V2(RC112), HiScript lll 1 st Strand cDNA Synthesis Kit(R312), Taq Pro Universal SYBR qPCR Master Mix(Q712)(Vazyme,China); riboFECT CP Transfection Kit(166 T)(C10511 - 05); Primer (Sangon,China); RIPA(P0013B) (Beyotime,China); BCA protein assay kit (WB6501) and ECL Luminescent Solution AB Solution (P2100)(NCM Biotech,China); Prestained Protein Marker II(G2058 - 250UL) (Servicebio,China); Recombinant Anti-NLRP3 antibody [EPR23094 - 1] (ab263899) (Abcam, UK); Recombinant Anti-GSDMD antibody [EPR19829] (ab210070) (Abcam, UK); Description: Caspase 1 Antibody—#AF5418 (Affinity Bioscience, CHN); Recombinant Anti-TMS1/ASC antibody [EPR23978 - 28] (ab283684) (Abcam, UK); Cleaved Caspase- 1 (Asp297) (D57 A2) Rabbit mAb #4199 (CST,US); Cleaved Caspase- 1 (ASP297) (D57 A2) Rabbit mab #4199 (CST,US) Recombinant Anti-cleaved N-terminal GSDMD antibody [EPR20829 - 408] (ab215203) (Abcam, UK); Recombinant Anti-IL- 1 beta antibody [EPR24895 - 116] (ab315084) (Abcam, UK); IL- 18 (D2 F3B) Rabbit mAb #54943 (CST,US); Recombinant Anti-Cdk6 antibody [EPR4515] (ab124821) (Abcam, UK); Recombinant Anti-Cdk4 antibody [EPR4513 - 32–7] (ab108357) (Abcam, UK); Recombinant Anti-Cyclin D1 antibody [EPR2241] C-terminal (ab134175) (Abcam, UK); Recombinant Anti-Cdk2 antibody [E304] (ab32147) (Abcam, UK); Recombinant Anti-Cyclin E1 antibody [EP435E] (ab33911) (Abcam, UK); Recombinant Anti-Caspase- 3 antibody [E87] (ab32351) (Abcam, UK); Recombinant Anti-Bcl- 2 antibody [EPR17509] (ab182858) (Abcam, UK); Recombinant Anti-Bax antibody [E63] (ab32503) (Abcam, UK); Anti-beta Actin antibody [AC- 15] (ab6276) (Abcam, UK); Goat Anti-Rabbit IgG H&L/HRP (bs- 0295G-HRP) (Bioss, China); Goat Anti-Mouse IgG H&L/HRP (bs- 0296G-HRP) (Bioss, China); Human IL- 1β ELISA Kit (PI305), Human IL- 18/IL- 1 F4 ELISA Kit (PI558) (Beyotime, China).

### Cell culture

Human esophageal epithelial cell line HET- 1 A (CL0139, Hunan Fenghui Biotechnology, China) was used in the present study. The cell line used have been authenticated with STR profile. When the cell confluence reaches 80%, the old culture medium is discarded. The cells are washed twice with 2 mL of PBS and then digested with 2 mL of 0.25% trypsin- 0.02% EDTA solution. Under a microscope, the cells are observed for about one minute until they round up, at which point 6 mL of complete culture medium is quickly added to stop the digestion. The cells are gently pipetted to collect them, and then centrifuged at 800 rpm for 5 min at 4 °C. After discarding the supernatant, the cells are counted and seeded into sterile culture flasks containing 10% FBS. The cells are cultured in an incubator at 37 °C with 5% CO_2_, and the medium is replaced every 2 days until the cells reach 80% confluence.

### Experimental grouping

Part 1 (Table 1): This part of the experiment involves inducing damage to human esophageal epithelial cells HET- 1 A using acid and bile salts to observe the changes in miR- 223 - 3p and the NLRP3 inflammasome pathway. The specific grouping is as follows.

**Table 1:** Experimental grouping for part one

Grouping	Specific Procedures
CK	The cells were maintained under standard culture conditions
model	The culture medium was adjusted to pH 4 using 1 mM HCl, followed by treatment with a combination of 0.25 mM glycochenodeoxycholic acid, 0.1 mM taurocholic acid sodium salt, 0.3 mM glycodeoxycholic acid sodium salt, and 0.1 mM taurodeoxycholic acid sodium salt for 6 h

The second part of the study focused on the regulatory effects of miR- 223 - 3p on NLRP3 and acid-induced injury in human esophageal epithelial cells HET- 1 A. The specific experimental groups were as follows (Table 2).

**Table 2:** Experimental groups for part 2

Grouping	Specific Procedures
model	The culture medium was adjusted to pH 4 using 1 mM HCl, followed by treatment with a combination of 0.25 mM glycochenodeoxycholic acid, 0.1 mM taurocholic acid sodium salt, 0.3 mM glycodeoxycholic acid sodium salt, and 0.1 mM taurodeoxycholic acid sodium salt for 6 h
OE-miR- 233 - 3p	Overexpression of miR- 223 - 3p combined with the same acid and bile salt treatment as described above
OE-miR- 233 - 3p-NC	Overexpression of miR- 223 - 3p-NC combined with the same acid and bile salt treatment as described above
Si-miR- 233 - 3p	Knockdown of miR- 223 - 3p combined with acid and bile salt treatment as described above
Si-miR- 233 - 3p-NC	Knockdown of miR- 223 - 3p-NC combined with the acid and bile salt treatment as described above

The third part involves the regulation of NLRP3 in acid-induced human esophageal epithelial cells HET- 1 A by miR- 223 - 3p, with specific experimental groups as follows (Table 3).

**Table 3:** Experimental grouping for part 3

Grouping	Specific Procedures
model	The culture medium was adjusted to pH 4 using 1 mM HCl, followed by treatment with a combination of 0.25 mM glycochenodeoxycholic acid, 0.1 mM taurocholic acid sodium salt, 0.3 mM glycodeoxycholic acid sodium salt, and 0.1 mM taurodeoxycholic acid sodium salt for 6 h
OE-miR- 233 - 3p	Overexpression of miR- 223 - 3p combined with the same acid and bile salt treatment as described above
OE-miR- 233 - 3p-NC	Overexpression of miR- 223 - 3p-NC combined with the same acid and bile salt treatment as described above
OE-miR- 233 - 3p + OE-NLRP3	Overexpression of miR- 223 - 3p combined with overexpression of NLRP3 under the same acid and bile salt conditions as described above
OE-miR- 233 - 3p + OE-NLRP3-NC	Overexpression of miR- 223 - 3p concomitant with overexpression of NLRP3-NC under the same acid and bile salt conditions as described above

### Cell transfection

1. miR- 223 - 3p Transfection For miR- 223 - 3p transfection, constructs of miR- 223 - 3p mimic, inhibitors, and NC were prepared. Inhibitor sequences were designed by targeting gene coding regions, with BLAST analysis ensuring specificity while excluding self-silencing regions. Mimics were designed based on full-length ORFs to maintain structural integrity. All sequences were experimentally validated for silencing/overexpression efficiency prior to application. The sequence of miR- 223 - 3p is TGTCAGTTTGTCAAATACCCCA, the inhibitor NC sequence is UCUACUCUUUCUAGGAGGUUGUGA, and the mimics NC sequences are UCACAACCUCCUAGAAAGAGUAGA and UCUACUCUUUCUAGGAGGUUGUGA. According to these sequences, corresponding mimic, inhibitor, and in-NC sequences were designed. Cells were counted, and 1 × 10^6^ cells were seeded per well in 6-well plates. Transfection was performed when cells reached approximately 50% confluence. Transfection reagents were prepared according to the table below: The final concentration of the transfection system in a 6-well plate was 50 nM with a volume of 2 mL per well. The medium was 1863 μL. The system included 120 μL of riboFECT CP buffer (v2), 5 μL of miRNA (v3), and 12 μL of riboFECT CP reagent (v4). After gently mixing v2, v3, and v4, the mixture was incubated at room temperature for 15 min to prepare the transfection complex. The transfection complex was then added dropwise to cells in complete medium without antibiotics, followed by gentle mixing. The culture plate was placed in a 37 °C, 5% CO_2_ incubator for cultivation.
2. NLRP3 Overexpression To construct the OE-NLRP3 lentiviral overexpression vector, the pLKO.1 with overexpression construct vector was chosen and synthesized by Suzhou GemGene Biotechnology Co., Ltd. The coding sequence (CDS) of NLRP3 gene (NM_001079821.3:128–3232) was inserted into this vector, which was then packaged into lentiviral expression vectors using a lentiviral packaging system. Cells were seeded into 6-well plates and cultured until reaching 80% confluence. Transfection reagents included diluted Lipofectamine 3000 and plasmid mixture. After incubation at room temperature for 15 min, the mixture was added to 293 T cells for transfection. The medium was changed to complete DMEM after 8 h, and virus-containing supernatant was collected 48 h later and filtered for storage. Lentiviral vector virus and Polybrene were added to cells in 6-well plates for infection. After 8 h, the medium was changed to complete culture medium for an additional 24 h, followed by selection in complete medium containing puromycin (PURO) to obtain stable cell lines overexpressing NLRP3.

### Flow cytometry analysis of cell apoptosis

Log-phase growing cells were seeded at 1 × 10^6^ cells/well in 6-well plates and cultured at 37 °C, 5% CO_2_ for 24 h until adherent. Cells were harvested and centrifuged at 1000 rpm for 5 min, followed by removal of the supernatant. Cells were resuspended in 1 mL PBS and centrifuged again as described above. The cell pellet was resuspended in 1X Annexin V Binding Solution to a final concentration of 1 × 10^6^ cells/mL. 100 μL of cell suspension was transferred to a new tube and incubated at room temperature in the dark with 5 μL Annexin V-FITC and 5 μL PI Solution for 15 min. Finally, 400 μL of 1X Annexin V Binding Solution was added, and the samples were analyzed within 1 h.

### Flow cytometric analysis of cell cycle

Cells were cultured in 6-well plates until reaching 70% confluence. Depending on experimental requirements, cells were treated and continued to culture. After treatment, cells were transferred to 1.5 mL centrifuge tubes and centrifuged at 200 g for 5 min. Cells were washed twice with PBS, each time centrifuged at 200 g for 5 min, and collected at 1–5 × 10^5^ cells. Cells were fixed in 1 mL pre-cooled 70% ethanol, gently mixed, and incubated at 4 °C for 4 h. After centrifugation at 200 g for 5 min, cells were resuspended in 1 mL pre-cooled PBS, centrifuged again, and supernatant gently removed to avoid cell loss, leaving approximately 50 μL PBS to disperse cells. Cells were stained with 0.5 mL propidium iodide staining solution, thoroughly resuspended, and incubated at 37 °C in the dark for 30 min. Stained samples were stored in the dark at 4 °C or on ice and analyzed within 24 h using a 488 nm wavelength for excitation.

### ELISA detection of IL- 1β and IL- 18 Levels

Cells in logarithmic growth phase were seeded at 1 × 10^6^ cells per well in 6-well plates and cultured at 37 °C with 5% CO_2_ for 24 h until they adhered. Cell culture supernatants were collected and centrifuged at 1000 × g for 20 min to obtain the supernatant for testing. Standards were diluted in a 96-well plate according to the instructions of the ELISA kit. To each well of the enzyme-labeled plate, 50 μL of standards and 50 μL of test samples were added and gently mixed. After sealing the plate with a cover film, it was incubated at 37 °C for 30 min. The concentrated washing solution was diluted 30 times with distilled water. After incubation, the liquid was discarded, and each well was filled with washing solution, left to stand for 30 s, discarded, tapped dry, and repeated five times. Then, 50 μL of enzyme-labeled reagent (excluding the blank wells) was added to each well. After sealing the plate with a cover film, it was incubated at 37 °C for 30 min, followed by a repeat of the washing steps. Subsequently, 50 μL each of color developer A and B were added to each well, gently mixed, and incubated at 37 °C in the dark for 15 min. Finally, 50 μL of stop solution was added to each well to terminate the reaction (turning blue to yellow). The absorbance (OD value) of each well was measured at a wavelength of 450 nm.

### MTT assay for determining cell IC_50_

Cells in logarithmic growth phase were seeded at 1 × 10^4^ cells per well in 96-well plates and allowed to adhere. To each well, 10 μL of MTT solution at a concentration of 0.5 mg/mL was added, and the plates were incubated for 4 h to allow MTT to be reduced to purple formazan. After incubation, 100 μL of DMSO was added to dissolve the formazan crystals, and the absorbance was measured to determine cell viability.

### EDU assay for detecting cell proliferation

Cells in logarithmic growth phase at 1 × 10^6^ cells were seeded in 6-well plates and treated according to experimental groups. The 2X EdU working solution was preheated to 37 °C, and an equal volume was added to each well to achieve a final EdU concentration of 1X, followed by incubation for 2 h. After removing the culture medium, cells were fixed with 1 mL of 4% paraformaldehyde for 15 min. Cells were then washed three times with 1 mL of washing solution for 3–5 min each and permeabilized with 1 mL of PBS containing 0.3% Triton X- 100 for 15 min. Click reaction mixture was prepared and added to each well after removing the washing solution. The plate was gently mixed and incubated in the dark for 30 min. After removing the reaction mixture, cells were washed three times for 5 min each. Subsequently, 1 mL of DAPI staining solution was added to each well and incubated for 5 min, followed by three washes for 5 min each. Fluorescence microscopy was performed using a 490 nm excitation filter. Cells were centrifuged at 450 × g for 5 min, resuspended in PBS, and 3–5 μL were pipetted onto slides, covered with coverslips, and examined under a fluorescence microscope.

### Western blot

Cells in logarithmic growth phase were seeded at 1 × 10^6^ cells per well in 6-well plates and cultured at 37 °C with 5% CO2 for 24 h post treatment. After removing the supernatant, cells were washed twice with pre-chilled PBS (0.5 mL per well) containing PMSF, followed by cell lysis using 0.5 mL of RIPA buffer at 4 °C with centrifugation at 12,000 × g for 5 min to collect the supernatant. Protein quantification was performed using the BCA method. BCA working solution was prepared, protein standards were diluted and added to a 96-well plate, and the volume was adjusted to 20 μL with dilution buffer. Samples were prepared by adding samples or supplementing with 20 μL of dilution buffer. Each well received 200 μL of BCA working solution, followed by incubation at 37 °C for 30 min, and absorbance was measured at 562 nm to draw a standard curve and calculate protein concentration. Samples were prepared by adding 5 × Loading Buffer and heating in a boiling water bath for 10 min. SDS-PAGE electrophoresis was performed using 12% separating gel and 5% stacking gel, adjusting the voltage for separation, and terminating electrophoresis. PVDF membrane was cut and activated with methanol, then soaked in transfer buffer before assembling the membrane sandwich for transfer under constant pressure. After transfer, membranes were washed with TBST and blocked for 30 min at 4 °C, followed by overnight incubation with primary antibodies in TBST. Membranes were washed with TBST and incubated with secondary antibodies at room temperature for 2 h, followed by another round of washing. Chemiluminescent reagents were mixed and applied to the PVDF membrane, and chemiluminescent imaging was performed using an imaging system. Image J software was used to analyze the optical density values, with relative expression calculated as the grayscale value of the target protein divided by that of the reference protein. The dilution ratios for antibodies used are listed in Table 4.

**Table 4:** The dilution of antibodies

Antibodies	Dilution (application)
NLRP3	1:1000 (WB)
ASC	1:1000 (WB)
caspase- 1	1:1000 (WB)
cleaved caspase- 1	1:1000 (WB)
GSDMD	1:1000 (WB)
GSDMD-NT	1:1000 (WB)
IL- 1β	1:1000 (WB)
IL- 18	1:1000 (WB)
CDK6	1:50,000
CDK4	1:50,000
cyclin D1	1:10,000
CDK2	1:1000
cyclin E1	1:1000
Caspase- 3	1:5000
Bcl- 2	1:2000
Bax	1:2000
β-actin	1:5000 (WB)
Goat Anti-Rabbit IgG H&L(HRP)	1:20,000 (WB)
Goat Anti-Mouse IgG H&L(HRP)	1:20,000 (WB)

### RT-qPCR

After RNA extraction, 500 ng of RNA was mixed with 5 × g DNA wiper mix and incubated at 42 °C for 2 min. Subsequently, 10 × RT Mix, Hiscript III Enzyme Mix, Oligo(dT)20 VN, Random hexamers, and RNase-free water were added, followed by incubation at 37 °C for 15 min and then 85 °C for 5 s to obtain cDNA, which was stored at − 20 °C. Real-time quantitative PCR was performed in a 20 μL reaction system, mixing fivefold diluted cDNA, nuclease-free water, 2 × Taq Pro Universal SYBR qPCR Master mix, forward primer, and reverse primer. The reaction was conducted in an 8-tube strip within a CFX96 Touch Real-Time PCR Detection System, with the following parameters: initial denaturation at 95 °C for 30 s, followed by 40 cycles of denaturation at 95 °C for 10 s, and annealing/extension at 60 °C for 10 s. A melting curve analysis was performed (95 °C for 15 s, 65 °C for 60 s, 95 °C for 15 s). The results were analyzed using the 2^−ΔΔCt^ method.

### Dual-luciferase reporter assay

Firstly, the sequence of miR- 223 - 3p was obtained from NCBI, and the binding site of miR- 223 - 3p with NLRP3 was predicted using TargetScan. Based on the predicted binding site, the miR- 223 - 3p/NLRP3 luciferase reporter vector was designed. The pmir-GLO vector was used to construct the reporter vector with the target gene fused with firefly luciferase and Renilla luciferase, where firefly luciferase (FLuc) served as the primary reporter gene and Renilla luciferase (RLuc) as the internal control (driven by the TK promoter). The dual-luciferase reporter plasmids were obtained from Shanghai Generay Biotech Co., Ltd. and divided into four groups: wild-type control, wild-type experimental, mutant control, and mutant experimental. Each group included the corresponding plasmid and miRNA mimics. In the mutant experimental group, the mutation site in the NLRP3 3'UTR was CGCUAUCUUUCUAUUAACUGACC, and the sequence of miR- 223 - 3p was ACCCCAUAAACUGUUUGACUGU. Subsequently, co-transfection experiments were conducted by co-transfecting the luciferase-tagged target gene and reference gene plasmids into HEK- 293 T cells for 48 h, with a ratio of 50:1 for the target gene plasmid to reference gene plasmid. The cells were then treated, including cell lysis, supernatant measurement, and measurement of firefly luciferase using the Dual-Luciferase Reporter Assay System. Finally, relative luciferase activity (RLU) was calculated based on the measured Flu values and Rlu values for each group.

### Data analysis

All experiments were performed in triplicate. Data were analyzed and plotted using GraphPad Prism 9 (Version 9.4.0). All data are presented as means ± SD. Inter-group statistical differences were assessed using T-tests or One-way ANOVA, with *P* < 0.05 considered statistically significant.

## Results

### Bile and acidic environment induce reflux esophagitis in HET- 1 A cells

**Fig. 1:**
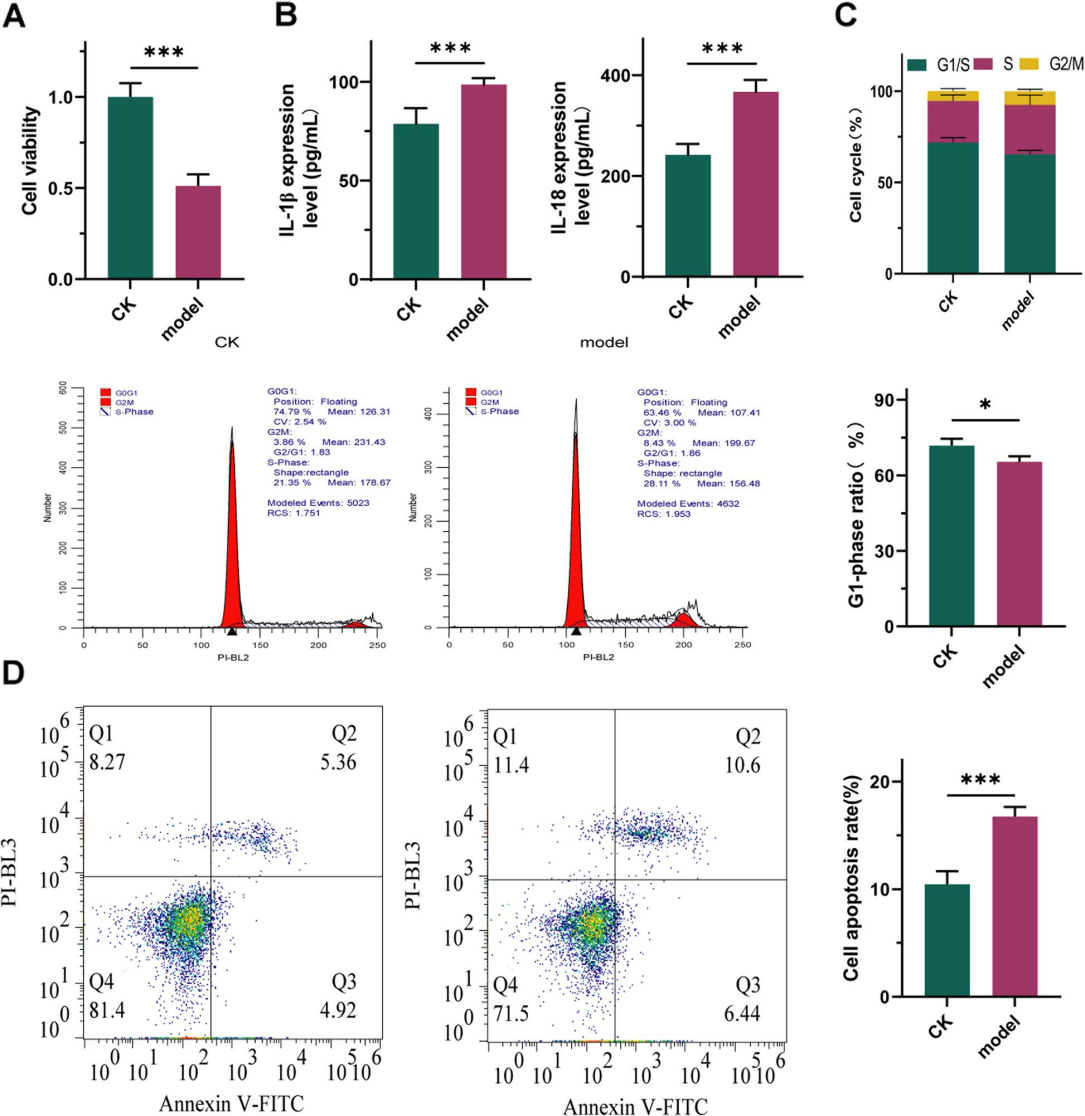
Induction of Reflux Esophagitis in HET- 1 A Cells by Bile and Acidic Environment. **A**: Changes in cell viability. **B**: Secretion levels of IL- 1β and IL- 18. **C**: Changes in cell cycle. **D**: Apoptosis of cells. *n* = 3; ***: *P* < 0.001

As depicted in Fig. 1, simulation of gastroesophageal reflux in vitro revealed a significant decrease in cell viability of HET- 1 A cells upon exposure to acidic and bile environments (Fig. 1A, *P* < 0.001). Concurrently, the secretion of IL- 1β and IL- 18 by HET- 1 A cells was markedly increased (Fig. 1B, *P* < 0.001), while the proportion of HET- 1 A cells in the G1 phase significantly decreased (Fig. 1C, *P* < 0.05). Moreover, the number of apoptotic cells significantly increased (Fig. 1D, *P* < 0.001). Taken together, these findings indicate that bile and acidic environments induce reflux esophagitis in HET- 1 A cells.

### Downregulation of miR- 223 - 3p contributes to NLRP3 activation in reflux esophagitis

**Fig. 2:**
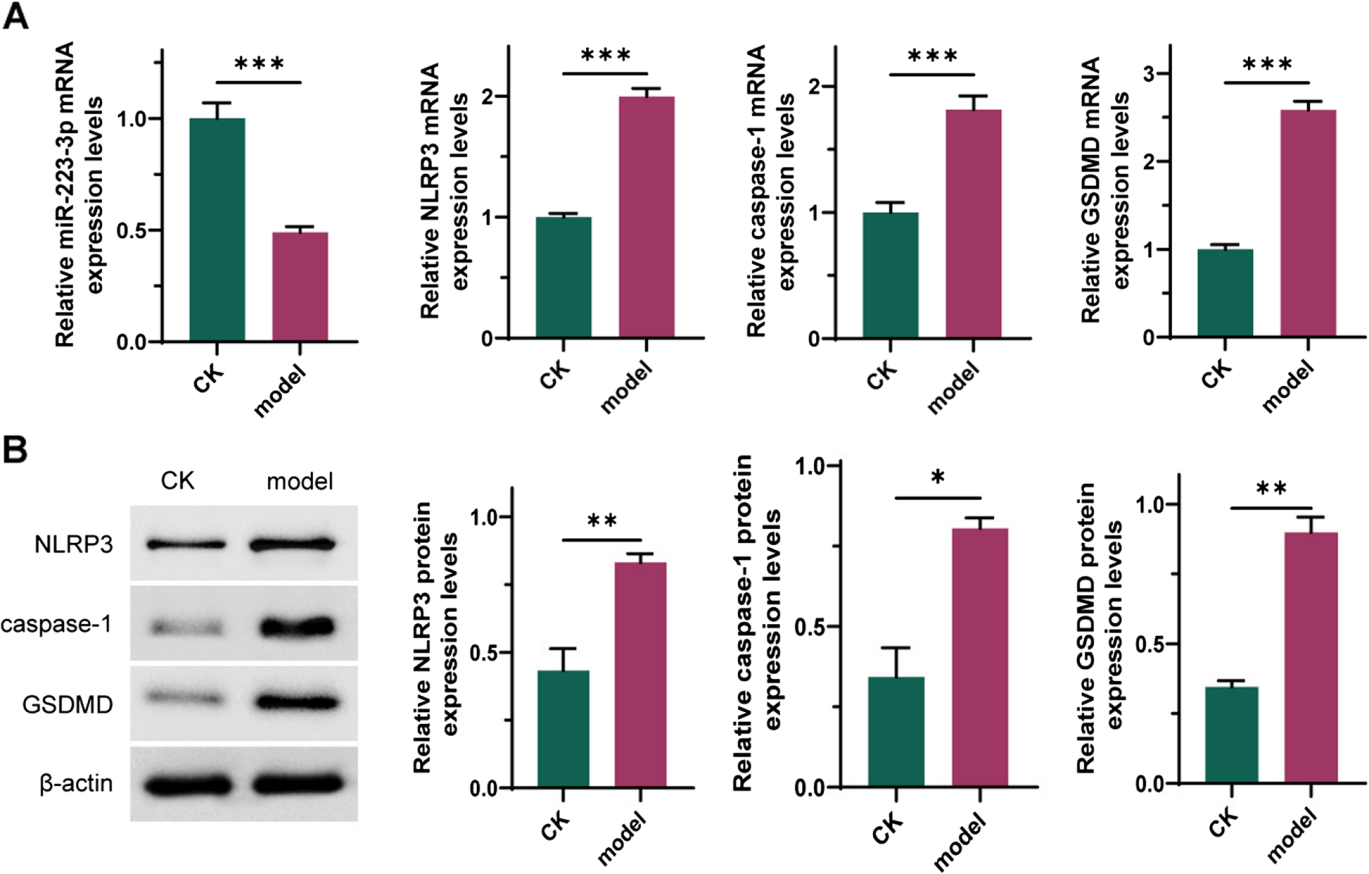
Downregulation of miR- 223 - 3p Contributes to NLRP3 Activation in Reflux Esophagitis. **A**: Expression levels of miR- 223 - 3p and NLRP3-related RNA. **B**: Expression levels of caspase- 1, NLRP3, and GSDMD proteins. *n* = 3; *: *P* < 0.05; **: *P* < 0.01; ***: *P* < 0.001

To investigate the mechanism by which bile and acidic environment mediate reflux esophagitis in HET- 1 A cells, as shown in Fig. 2, we measured the expression levels of miR- 223 - 3p and NLRP3-related proteins. The results revealed a significant decrease in miR- 223 - 3p expression during the development of reflux esophagitis in HET- 1 A cells (Fig. 2A, *P* < 0.001), accompanied by significant increases in the expression levels of caspase- 1, NLRP3, and GSDMD (Fig. 2A-B, *P* < 0.05). These findings suggest that the downregulation of miR- 223 - 3p and subsequent NLRP3 activation may be involved in the pathogenesis of reflux esophagitis. However, further investigations are needed to elucidate the specific mechanisms involved.

### Upregulation of miR- 223 - 3p inhibits the development of bile and acid-induced reflux esophagitis

**Fig. 3:**
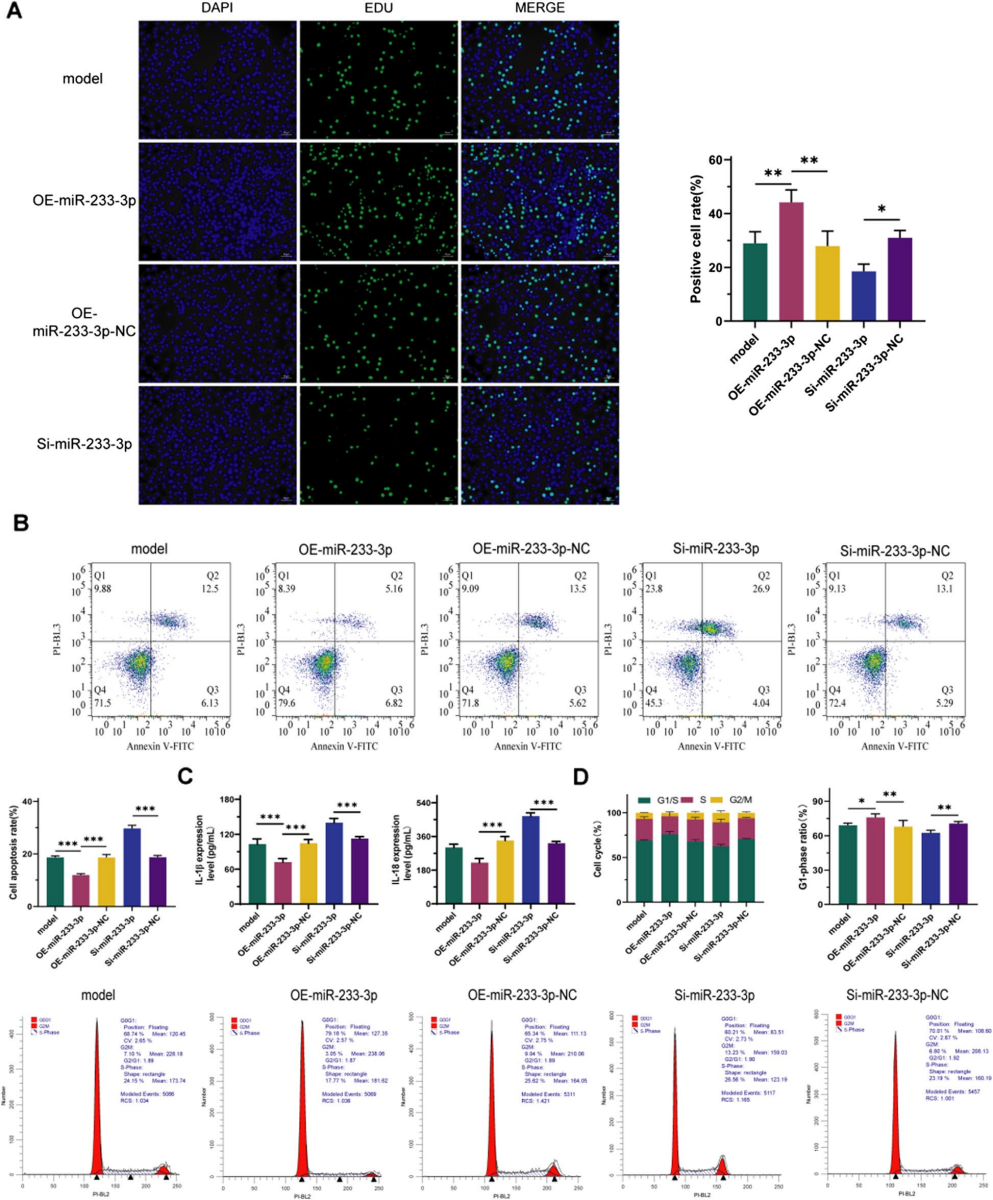
Upregulation of miR- 223 - 3p inhibits bile and acid-induced reflux esophagitis. **A**: Changes in cell proliferation. **B**: Changes in cell apoptosis. **C**: Levels of IL- 1β and IL- 18 secretion. **D**: Changes in cell cycle. *n* = 3; *: *P* < 0.05; **: *P* < 0.01; ***: *P* < 0.001

To validate the role of miR- 223 - 3p in the development of bile and acid-induced reflux esophagitis, we manipulated miR- 223 - 3p expression levels in HET- 1 A cells. As shown in Fig. 3, compared with the model group, overexpression of miR- 223 - 3p significantly increased the proliferation rate and the proportion of cells in G1 phase in HET- 1 A cells (Fig. 3A, 3D, *P* < 0.05), and markedly reduced the number of apoptotic cells as well as the secretion of IL- 1β and IL- 18 (Fig. 3B-C, *P* < 0.001). Conversely, these effects were reversed and exacerbated upon miR- 223 - 3p knockdown, indicating that upregulation of miR- 223 - 3p can inhibit the occurrence of bile and acid-induced reflux esophagitis. This suggests a potential therapeutic target for the management of this condition.

### miR- 223 - 3p regulates the activation of NLRP3

**Fig. 4:**
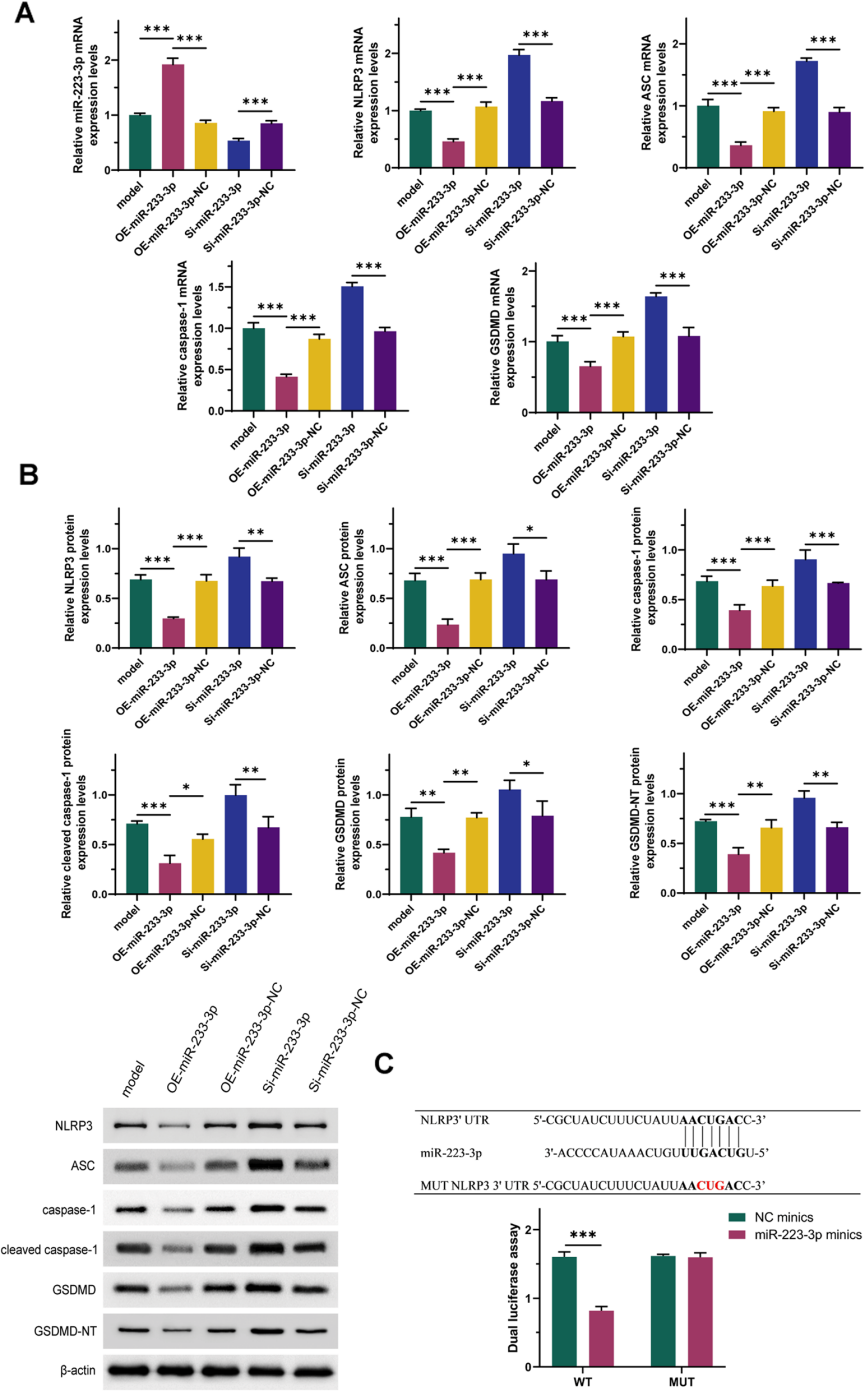
miR- 223 - 3p regulates the activation of NLRP3. **A**: Expression levels of miR- 223 - 3p and NLRP3 activation-related RNAs. **B**: Expression levels of NLRP3 activation-related proteins. **C**: Dual-luciferase reporter assay results. *n* = 3; *: *P* < 0.05; **: *P* < 0.01; ***: *P* < 0.001

Previous experiments have indicated that miR- 223 - 3p regulates the secretion of IL- 1β and IL- 18, which are products activated by NLRP3. Therefore, we hypothesized that miR- 223 - 3p may also regulate the activation of NLRP3. To test this hypothesis, we conducted the following experiments. The results showed that compared to the model group, overexpression of miR- 223 - 3p significantly reduced the expression of ASC, caspase- 1, cleaved caspase- 1, NLRP3, GSDMD, and GSDMD-NT (Fig. 4A-B, *P* < 0.001). Conversely, this effect was reversed and exacerbated upon miR- 223 - 3p knockdown. Additionally, dual-luciferase reporter assays demonstrated a direct targeting relationship between miR- 223 - 3p and NLRP3 (Fig. 4C). Taken together, these results indicate that miR- 223 - 3p regulates the activation of NLRP3.

### miR- 223 - 3p inhibits reflux esophagitis by targeting the NLRP3 inflammasome

**Fig. 5:**
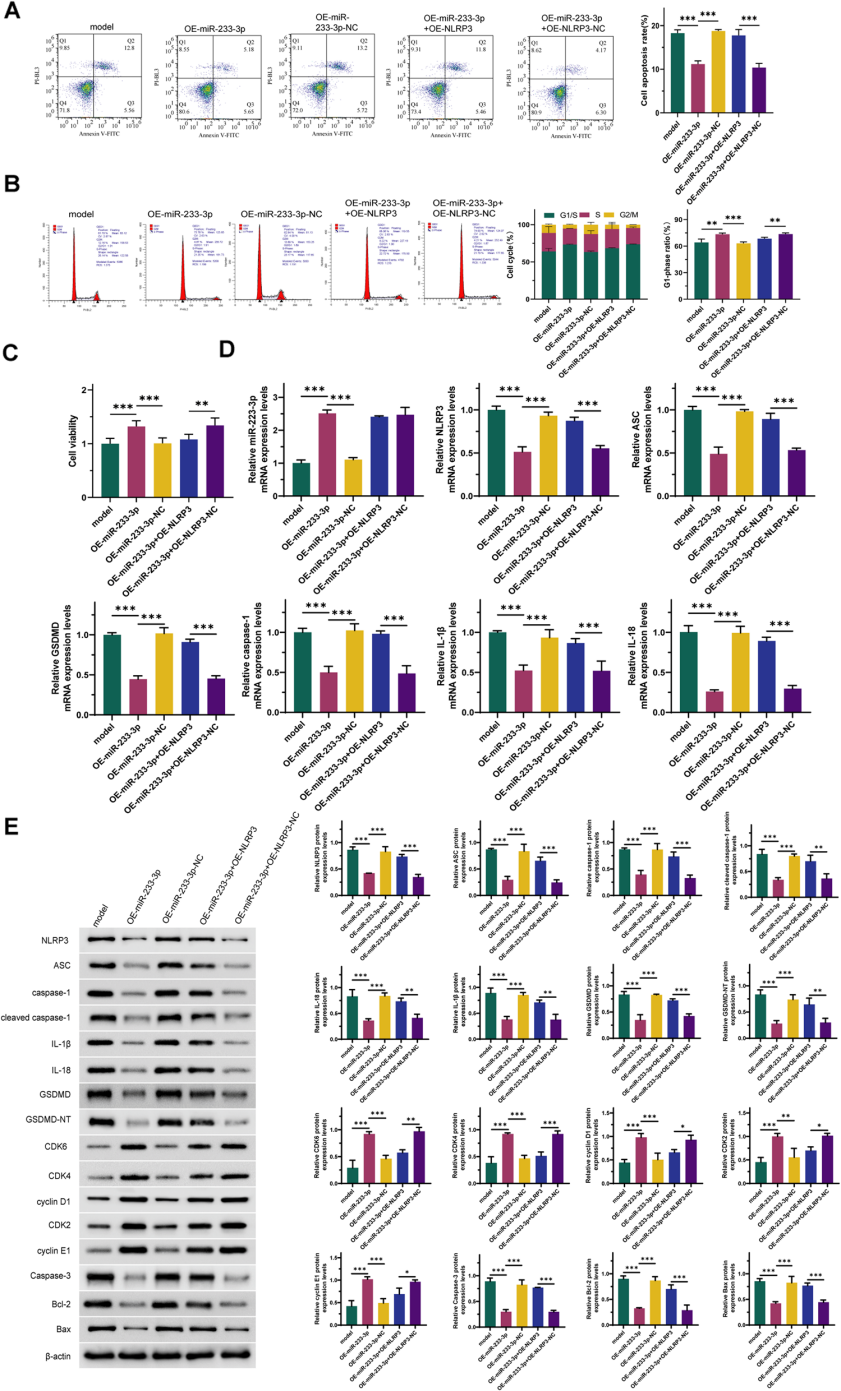
miR- 223 - 3p inhibits reflux esophagitis by targeting the NLRP3 inflammasome. **A**: Changes in cell apoptosis. **B**: Changes in cell cycle. **C**: Changes in cell viability. **D**: Expression levels of miR- 223 - 3p and NLRP3 activation-related RNAs. **E**: Expression levels of NLRP3 activation-related proteins, cell cycle-related proteins, and cell apoptosis-related proteins. *n* = 3; *: *P* < 0.05; **: *P* < 0.01; ***: *P* < 0.001

Based on previous experimental results, we hypothesized that miR- 223 - 3p may influence the development of reflux esophagitis by regulating the activation of the NLRP3 inflammasome. To validate this hypothesis, we conducted the following experiments. As shown in Fig. 5, compared with the model group, overexpression of miR- 223 - 3p alone improved bile and acid-induced reflux esophagitis and reduced the expression levels of NLRP3 inflammasome activation-related proteins. This result is consistent with our previous findings, demonstrating good reproducibility and reliability. Notably, compared to the OE-miR- 223 - 3p group, co-overexpression of miR- 223 - 3p and NLRP3 significantly increased the number of apoptotic cells (Fig. 5A, *P* < 0.001) and significantly elevated the expression levels of NLRP3, ASC, caspase- 1, cleaved caspase- 1, IL- 1β, IL- 18, GSDMD, GSDMD-NT, CDK6, CDK4, cyclin D1, CDK2, cyclin E1, caspase- 3, Bcl- 2, and Bax (Fig. 5D-E, *P* < 0.001). Additionally, it decreased the number of cells in the G1 phase and cell viability (Fig. 5B-C, *P* < 0.001). These findings suggest that NLRP3 overexpression can reverse the ameliorative effects of miR- 223 - 3p on reflux esophagitis, indicating that miR- 223 - 3p inhibits the development of reflux esophagitis by targeting the NLRP3 inflammasome.

## Discussion

This study delves into the mechanism by which miR- 223 - 3p inhibits reflux esophagitis through targeting the NLRP3 inflammasome, thereby enriching our understanding of the pathophysiology of reflux esophagitis. Our results indicate a significant downregulation of miR- 223 - 3p in the reflux esophagitis model, while the expression of the NLRP3 inflammasome and its related inflammatory factors were significantly upregulated. Through in vitro experiments, we validated that overexpression of miR- 223 - 3p significantly inhibits the activation of the NLRP3 inflammasome, thereby reducing cell apoptosis, promoting cell cycle progression, and enhancing cell viability. These findings provide experimental evidence for miR- 223 - 3p as a potential therapeutic target for reflux esophagitis and are discussed in conjunction with current research.

The NLRP3 inflammasome plays a crucial role in various inflammation-related diseases, such as arthritis, Alzheimer's disease, and inflammatory bowel disease [30–32], while miR- 223 - 3p has been shown to inhibit its expression by targeting NLRP3 mRNA [33]. Our study found that miR- 223 - 3p is significantly downregulated in the reflux esophagitis model, whereas NLRP3 and its downstream effectors, such as caspase- 1, IL- 1β, and IL- 18, are significantly upregulated. Pyroptosis, mediated by GSDMD, is characterized by NLRP3 inflammasome activation. NLRP3 inflammasome forms a complex upon detecting pathogen- or damage-associated molecular patterns (PAMPs/DAMPs), which activates caspase- 1. Caspase- 1 cleaves GSDMD to release its N-terminal pore-forming fragment (GSDMD-N). Oligomerized GSDMD-N creates transmembrane pores, disrupting osmotic balance and triggering release of IL- 1β and IL- 18 [34]. Therefore, this results suggest that miR- 223 - 3p plays a critical role in regulating the inflammatory response and pyroptosis. Previous studies have also demonstrated that miR- 223 - 3p has regulatory functions in various inflammatory and immune responses, providing a theoretical basis for our findings. For instance, Neudecker et al. reported that miR- 223 ameliorates intestinal inflammation in mice by regulating NLRP3 [21], studies have also demonstrated that miR- 223 - 3p targets and regulates the IL- 6 receptor subunit beta, thereby alleviating TNF-α-associated inflammatory responses [35]. Besides IL- 1β and IL- 18, these inflammatory cytokines may also be affected by miR- 223 - 3p, which will also serve as one of the directions for our subsequent research.

The G1/S cell cycle transition has been demonstrated in numerous studies to be closely associated with cellular viability. S-phase kinase-associated protein- 2 (Skp2), a pivotal regulator of G1/S phase transition, promotes ubiquitination-mediated degradation of cell cycle inhibitory proteins such as p27 Kip1 in human esophageal epithelial cells, thereby facilitating cell cycle progression from G1 to S phase. This regulatory mechanism is essential for maintaining precise control over DNA replication and mitotic division. Notably, under pathological conditions associated with impaired proliferative capacity, diminished Skp2 expression disrupts G1/S phase transition in human esophageal epithelial cells, resulting in cell cycle arrest and compromised regenerative potential, enhanced cellular viability has been shown to facilitate this critical phase transition during cell cycle progression [36, 37]. In this research, it is shown that overexpression of miR- 223 - 3p can significantly reduce apoptosis in HET- 1 A cells induced by acid and bile salts, increase the proportion of cells in the G1/S phase, and significantly enhance cell viability. Overexpression of NLRP3 can reverse the protective effects of miR- 223 - 3p, further confirming that miR- 223 - 3p reduces cell apoptosis and promotes G1/S cell cycle progression by inhibiting NLRP3 inflammasome activation. Under hyperhomocysteinemic conditions, activation of inflammatory mediators markedly impairs G1/S phase transition by suppressing Cyclin A transcription. Furthermore, the inflammatory microenvironment induces aberrant expression of cell cycle regulatory proteins, including HMGA2, CCNA2, and CCNB2, which collectively disrupt the molecular machinery governing cell cycle progression [38]. These alterations highlight a critical link between inflammatory signaling and cell cycle dysregulation. The finding in the research is consistent with previous studies, emphasizing the critical role of miR- 223 - 3p in regulating cell cycle, cell viability and inflammation progress of HET- 1 A with inflammation [39].

Reflux esophagitis is characterized by esophageal mucosal damage caused by gastric acid and bile salts reflux, often accompanied by a significant inflammatory response [40]. Current treatments primarily focus on inhibiting gastric acid secretion and improving esophageal mucosal protection but are limited by effectiveness and side effects. Our study found that miR- 223 - 3p can significantly ameliorate the inflammatory response in reflux esophagitis by targeting the NLRP3 inflammasome, providing a new therapeutic approach for reflux esophagitis. By increasing the expression of miR- 223 - 3p, it is possible to effectively inhibit the activation of the NLRP3 inflammasome, reduce the release of inflammatory factors, and thereby alleviate tissue damage [41]. For example, Lv et al. discovered that XIST inhibition alleviates renal inflammation and oxidative damage induced by calcium oxalate nephrolithiasis through the miR- 223/NLRP3 pathway [42].

This study further elucidates the molecular mechanism of miR- 223 - 3p, specifically by directly targeting NLRP3 mRNA, reducing the expression of NLRP3 protein, and subsequently inhibiting the assembly and activation of the inflammasome. Activation of the NLRP3 inflammasome typically requires multiple signaling steps, including primary signals (such as NF-κB pathway activation) and secondary signals (such as potassium efflux and mitochondrial damage) [43]. Our results suggest that miR- 223 - 3p may exert its inhibitory effect by intervening at key nodes within these steps. For instance, studies have shown that the miR- 223/TLR4/MyD88-NF-κB pathway inhibits hippocampal inflammatory responses in mice subjected to chronic unpredictable mild stress (CUMS) [44], it has also been reported that miR- 223 targets NF-κB and IRAK1 in macrophages. However, miR- 223 - 3p mediates the transition from chronic inflammation to gastric carcinogenesis through the NF-κB signaling pathway, due to the identification of a conserved NF-κB binding site within the promoter region of miR- 223 - 3p, and miR- 223 has no effect on the expression of NF-κB in the gastric cancer cells [45]. This opposite regulatory relationship suggests that there might be two different regulatory mechanisms in these two types of cells, which is even more worthy of further exploration. Additionally, studies have demonstrated that miR- 223 - 3p negatively regulates the expression of E2 F1 and STAT3 in chronic reflux esophagitis [46]. As a cell cycle regulator, E2 F1 modulates immune functions by controlling cell cycle-related genes such as CCNE1 [47]. E2 F1 could interact with inflammatory signaling pathways, including STAT3, potentially influencing the immune microenvironment through regulation of pro-inflammatory cytokines (e.g., IL- 6, IL- 1β) [48]. Furthermore, emerging evidence indicates that in addition to the NLRP3 inflammasome, TNF-α induced by the AIM2 inflammasome can upregulate miR- 223 - 3p expression, thereby suppressing NF-κB signaling [49]. These findings suggest that other inflammasomes may similarly exploit miR- 223 - 3p as a downstream effector to influence inflammatory cascades, and miR- 223 - 3p may exert multifaceted regulatory effects in reflux esophagitis, involving both immune- and inflammation-associated gene pathways.

Although this study has verified the anti-inflammatory effects of miR- 223 - 3p in vitro, its effects and mechanisms in vivo still require further investigation. Studies have compared various in vivo modeling methods (including indicators such as modeling duration, post-modeling survival rate, and modeling success rate). The results demonstrated that the rat model established through cardiotomy plus external pyloric ligation exhibited superior performance, suggesting this approach could serve as a valuable reference for further in vivo modeling experiments [50]. Additionally, the regulatory mechanisms of miR- 223 - 3p expression and its interactions with other inflammation-related molecules need to be further explored. Notably, miR- 223 - 3p may exhibit divergent effects in vivo. In a murine model of allergic rhinitis (AR), overexpression of miR- 223 - 3p exacerbated nasal mucosal epithelial shedding, eosinophil infiltration, and mucosal edema, indicating aggravation of AR-related inflammation. Mechanistically, miR- 223 - 3p was reported to suppress FBXW7 expression, thereby attenuating FBXW7-mediated inhibition of eosinophil degranulation. This led to increased release of pro-inflammatory factors (e.g., ECP, MBP, EPO) and amplified inflammatory responses. These findings contrast with the canonical anti-inflammatory role of miR- 223 - 3p, highlighting its context-dependent regulatory versatility in vivo.

Some research also reveal the expression of miR- 223 - 3p gradually decreases as reflux esophagitis progresses from the acute to the chronic stage, as it is deregulated in many inflammation-related disorders including a mouse inflammatory bowel disease model [46]. For example, elevated serum levels of miR- 223 have been observed in patients with chronic hepatitis B, while its expression is downregulated in hepatocellular carcinoma (HCC), suggesting a potential shift from initial upregulation during infection to subsequent downregulation in chronic liver injury or carcinogenesis. The authors hypothesize that epigenetic silencing in HCC tissues may account for miR- 223 downregulation, concurrently demonstrating its tumor-suppressive function [51]. Furthermore, in murine models of chronic kidney disease, miR- 223 levels display paradoxical alterations: increased in aortic tissues yet decreased in serum (particularly at advanced stages). It is suggested that elevated aortic miR- 223 correlates with vascular calcification, while reduced serum levels may result from enhanced excretion or consumption (though exact mechanisms remain to be elucidated) [52]. Collectively, these findings highlight the recurrent dysregulation of miR- 223 in chronic pathological conditions. According to our speculation, during acute inflammation or early infection phases, miR- 223 upregulation typically manifests its anti-inflammatory properties, conversely, its abnormal expression in chronic stages likely represents a compensatory feedback mechanism against persistent inflammation rather than direct inflammatory consequences. This regulatory pattern underscores the complex dual roles of miR- 223 in different disease contexts. Therefore, future research should further validate the role of miR- 223 - 3p in animal models and clinical samples and explore its feasibility and safety as a therapeutic target. Particularly, considering the multi-target characteristics of miRNAs, it is necessary to thoroughly evaluate their potential side effects and off-target effects in specific applications. For example, Jiao et al. pointed out that miR- 223 may have different regulatory effects under different pathological conditions [53].

## Conclusion

This study has validated the protective role of miR- 223 - 3p in reflux esophagitis through a series of experiments and revealed its mechanism of action in inhibiting the inflammatory response by targeting the NLRP3 inflammasome. The identification of miR- 223 - 3p as a potential therapeutic target for reflux esophagitis provides new insights and directions for the treatment of this disease. Future research should further verify its effects in vivo and explore its clinical application potential as a therapeutic target.

## Supplementary Information

Supplementary Material 1.

## Data Availability

The original data involved in the present study can be provided under reasonable request.
