# Supplementary figures and images for: Investigation of the mechanism by which miR-223-3p inhibits reflux esophagitis through targeting the NLRP3 inflammasome
Source: BMC Gastroenterol. 2025 May 13;25:365. doi: 10.1186/s12876-025-03836-9 (PMC12070713; doi:10.1186/s12876-025-03836-9)

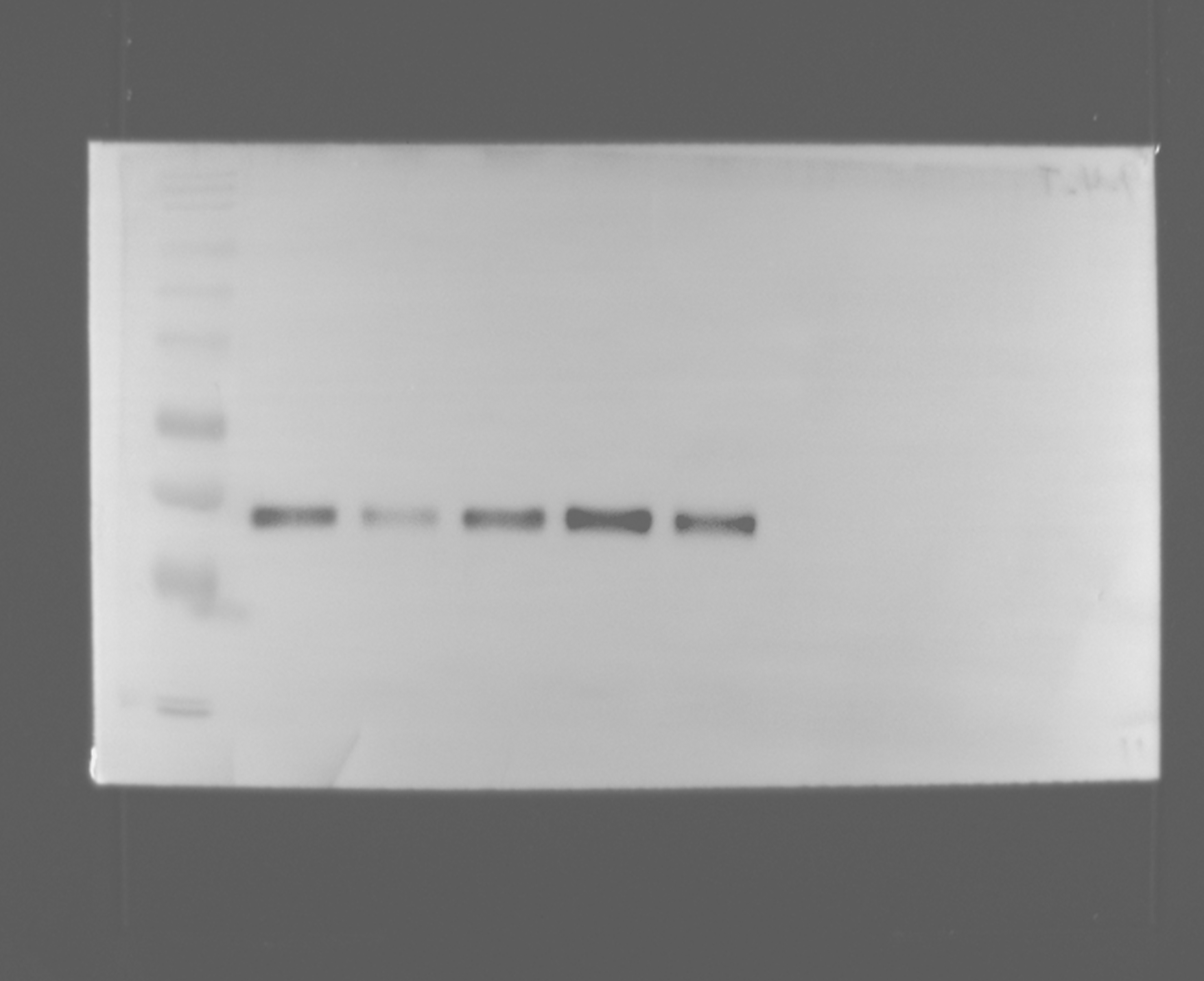

Supplement: Supplementary file 1 — Supplementary Material 1. [file 12876_2025_3836_MOESM1_ESM.zip › full uncropped Gels and Blots image/ASC 22KDa -1.tif]

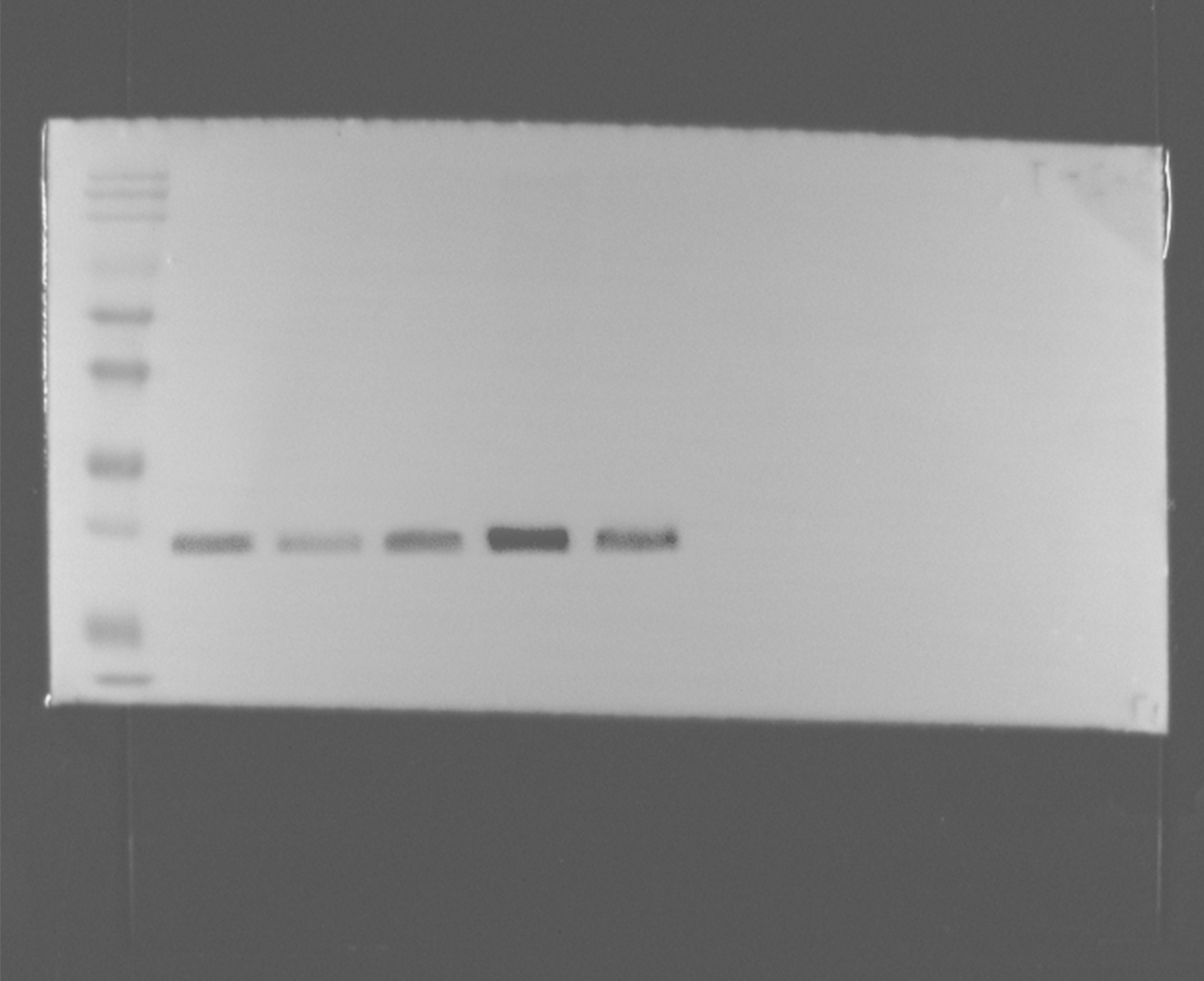

Supplement: Supplementary file 1 — Supplementary Material 1. [file 12876_2025_3836_MOESM1_ESM.zip › full uncropped Gels and Blots image/ASC 22KDa -2.tif]

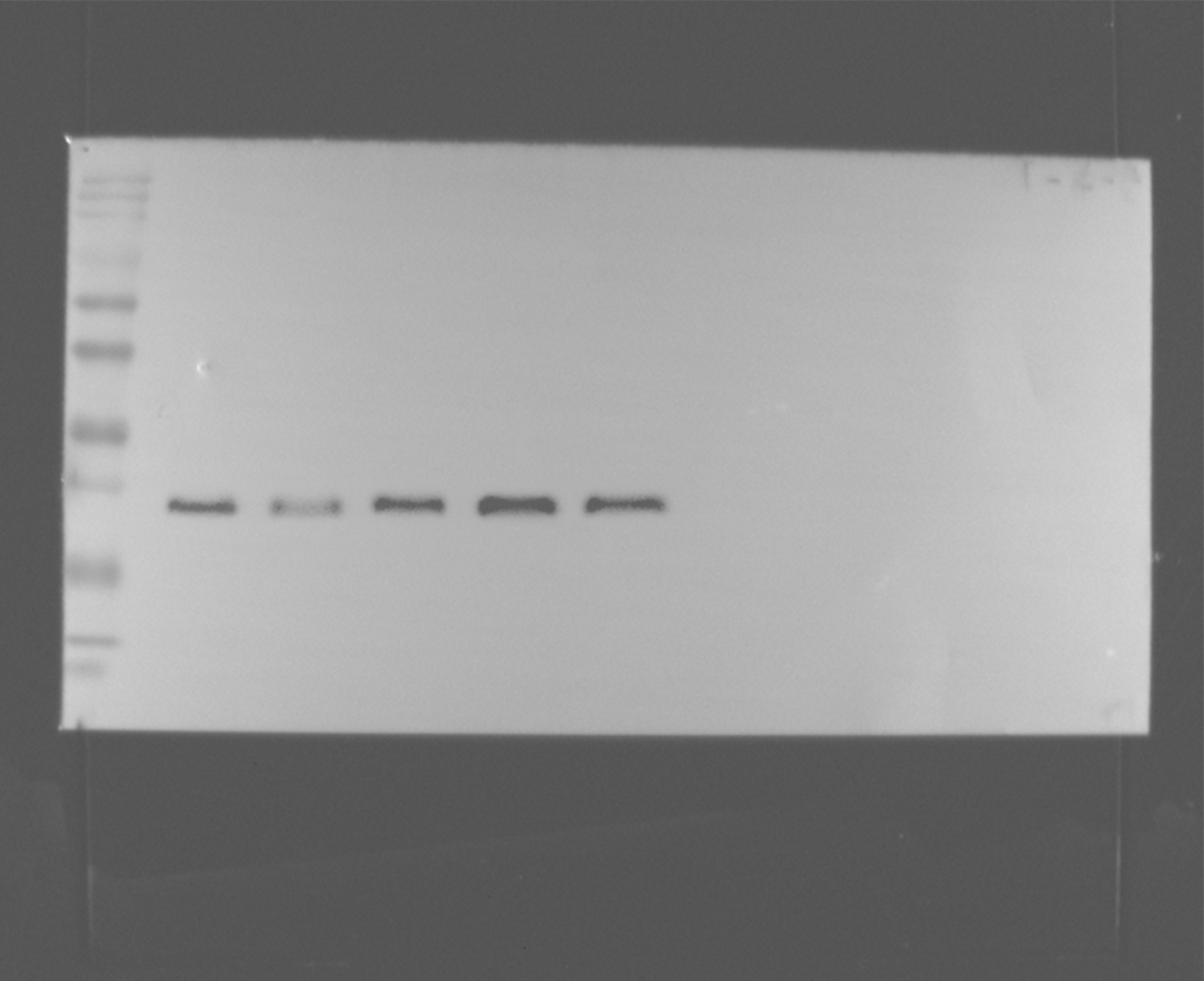

Supplement: Supplementary file 1 — Supplementary Material 1. [file 12876_2025_3836_MOESM1_ESM.zip › full uncropped Gels and Blots image/ASC 22KDa -3.tif]

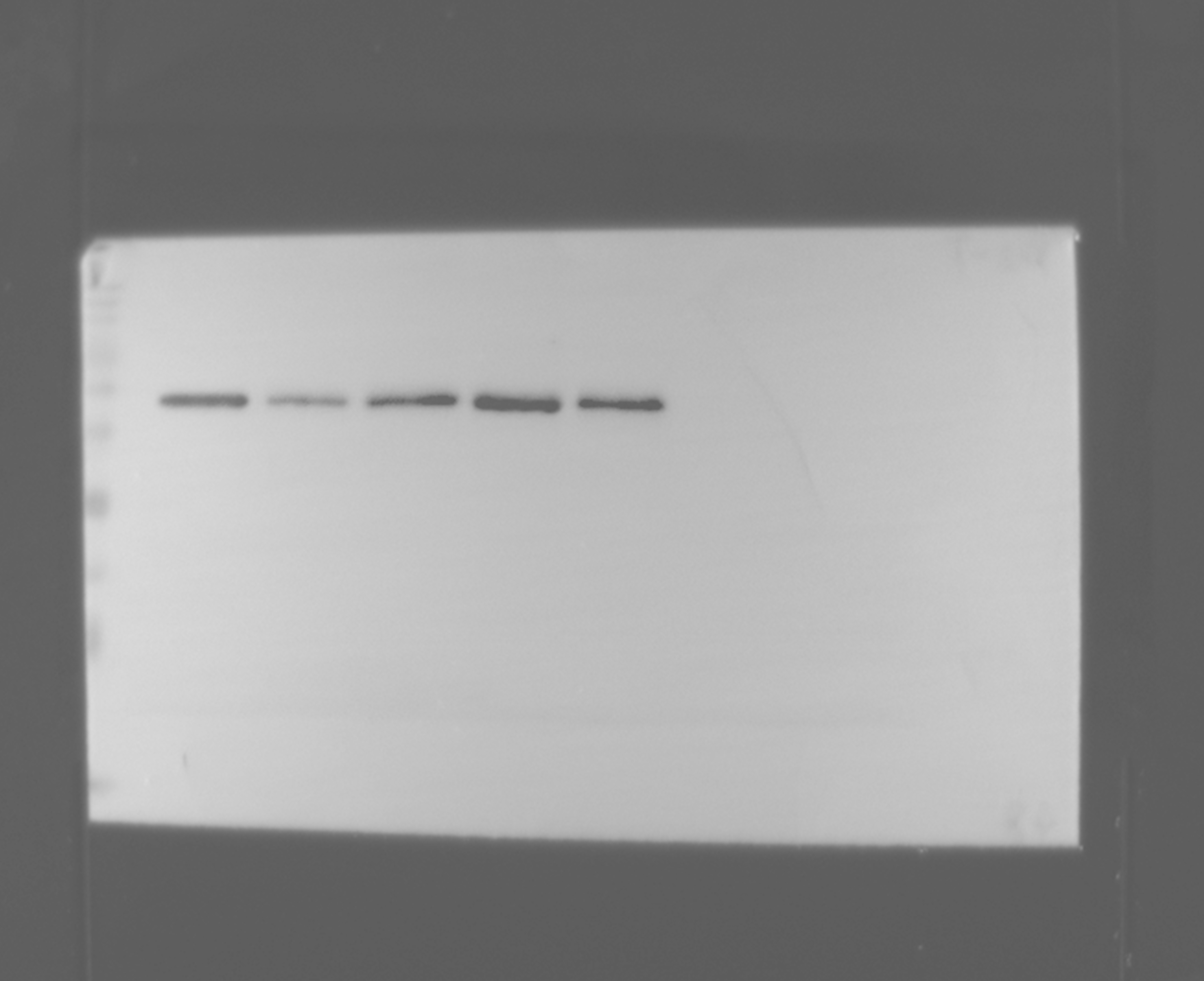

Supplement: Supplementary file 1 — Supplementary Material 1. [file 12876_2025_3836_MOESM1_ESM.zip › full uncropped Gels and Blots image/GSDMD 52KDa -1.tif]

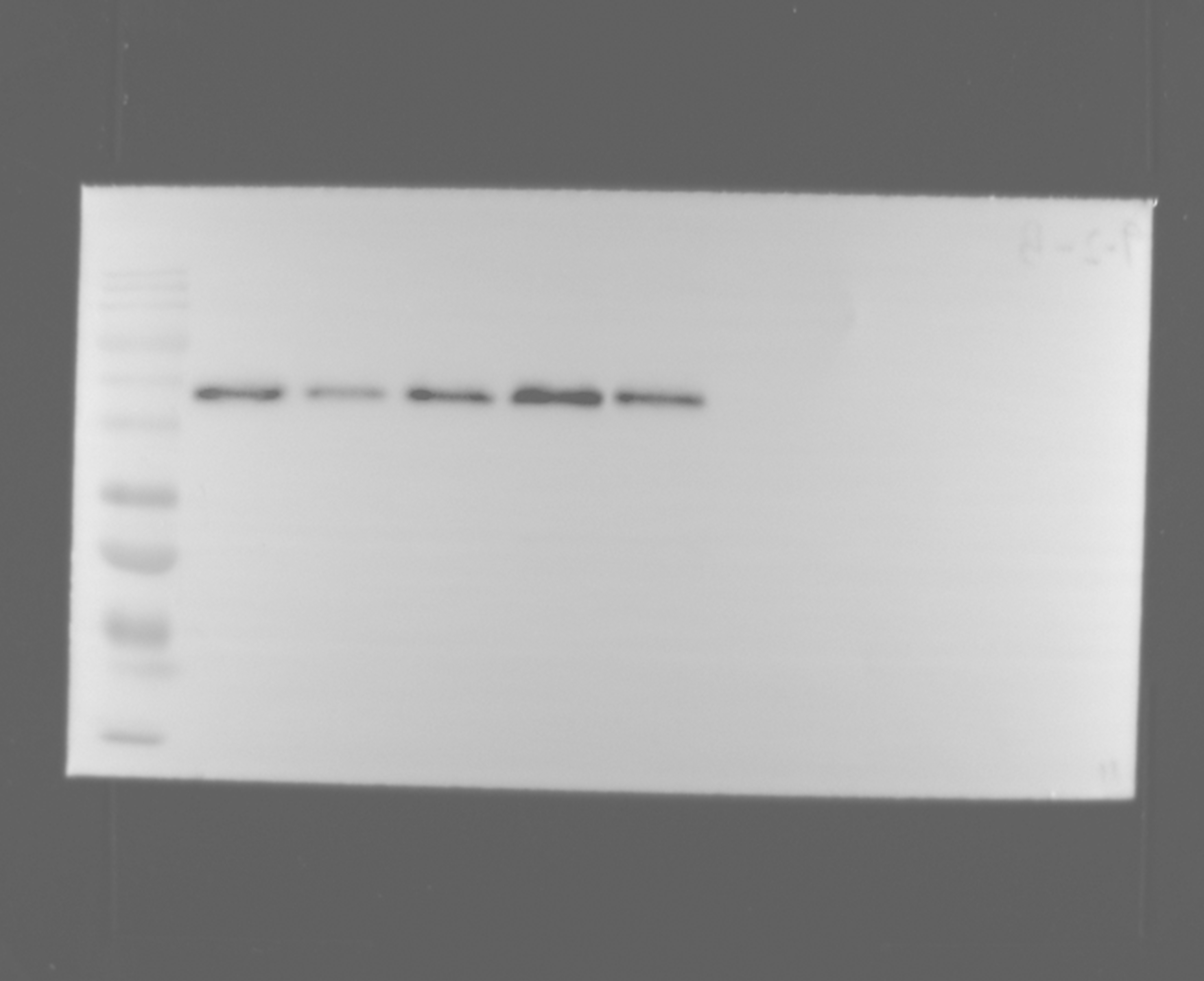

Supplement: Supplementary file 1 — Supplementary Material 1. [file 12876_2025_3836_MOESM1_ESM.zip › full uncropped Gels and Blots image/GSDMD 52KDa -2.tif]

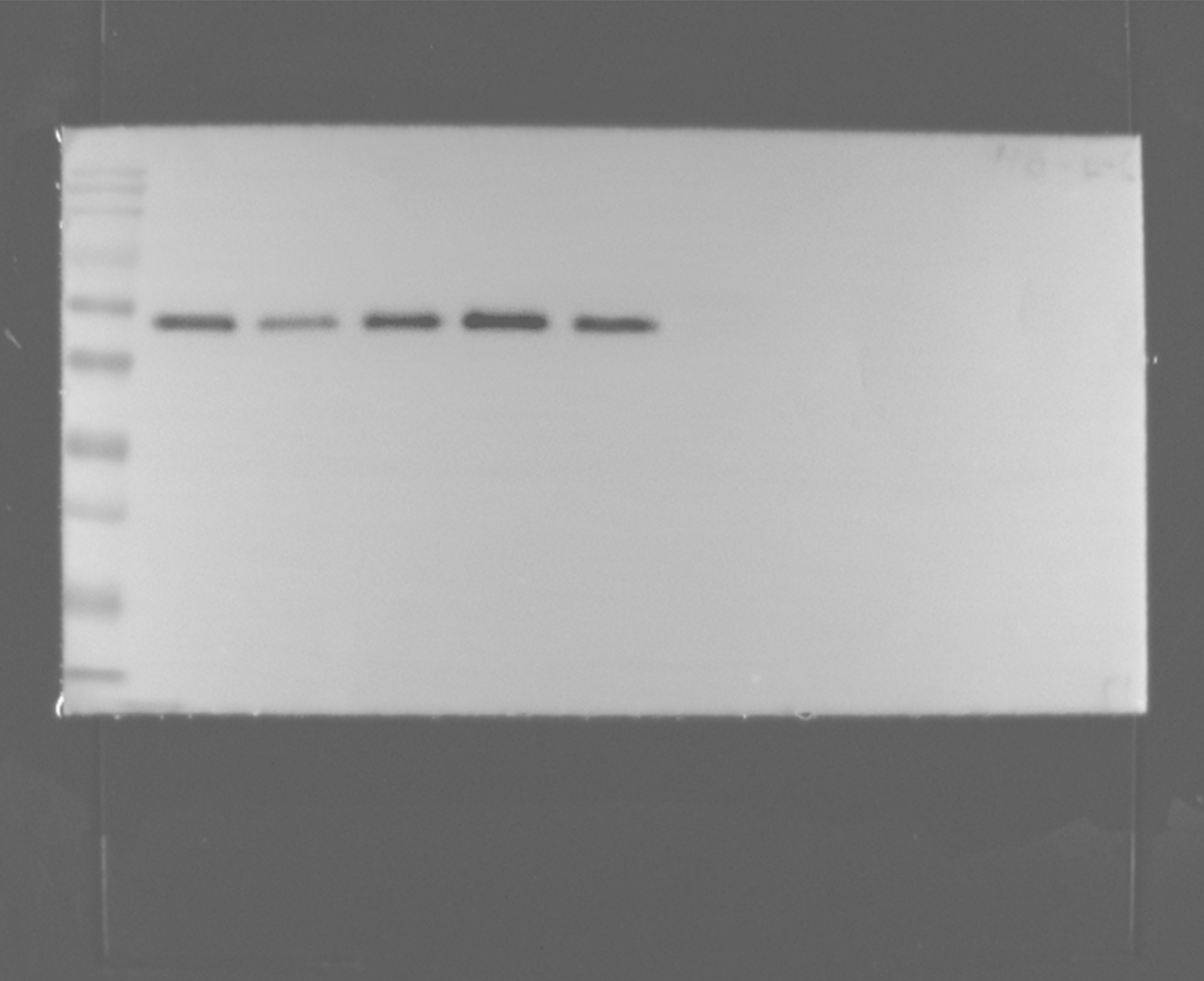

Supplement: Supplementary file 1 — Supplementary Material 1. [file 12876_2025_3836_MOESM1_ESM.zip › full uncropped Gels and Blots image/GSDMD 52KDa -3.tif]

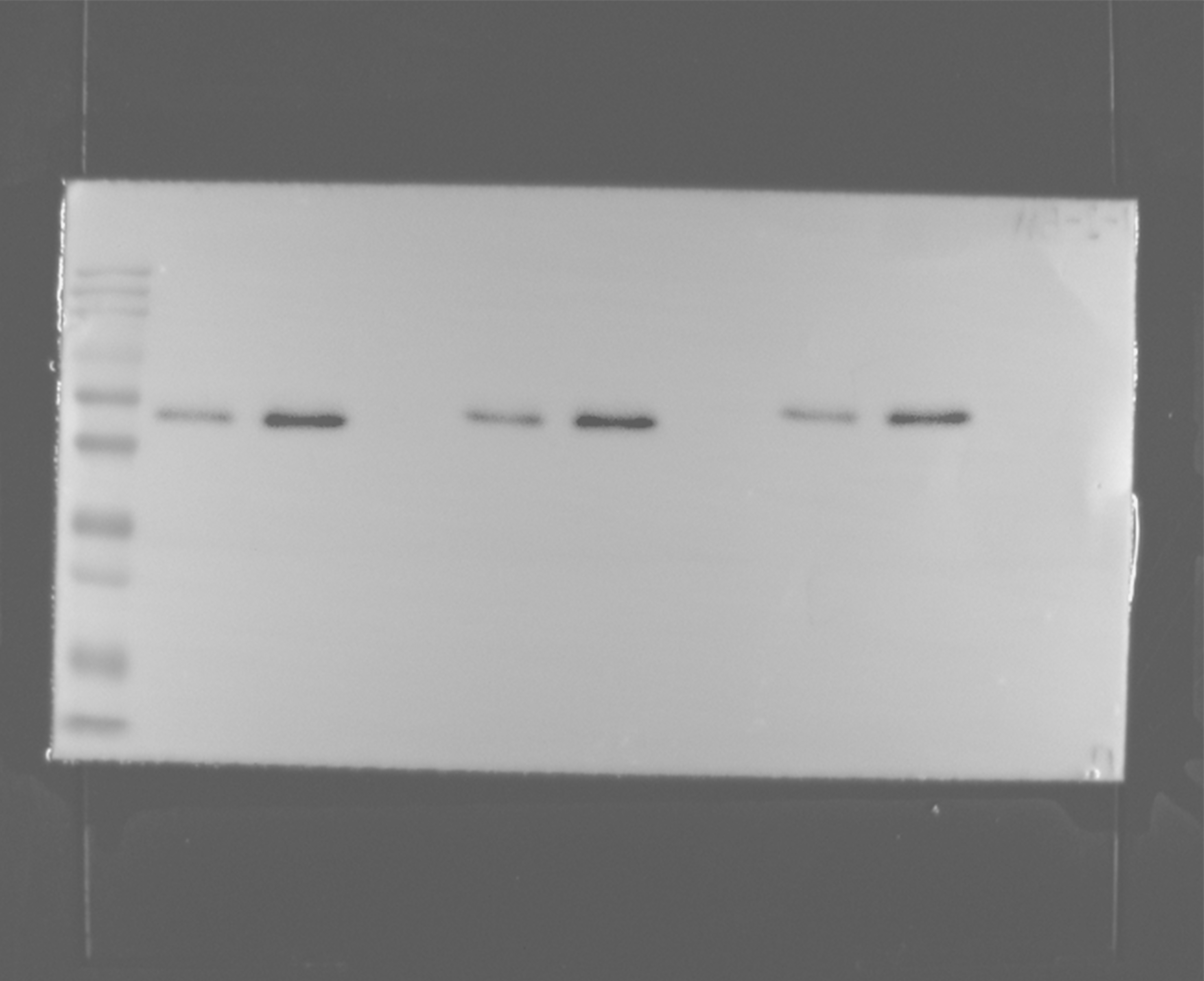

Supplement: Supplementary file 1 — Supplementary Material 1. [file 12876_2025_3836_MOESM1_ESM.zip › full uncropped Gels and Blots image/GSDMD 52KDa 01-03.tif]

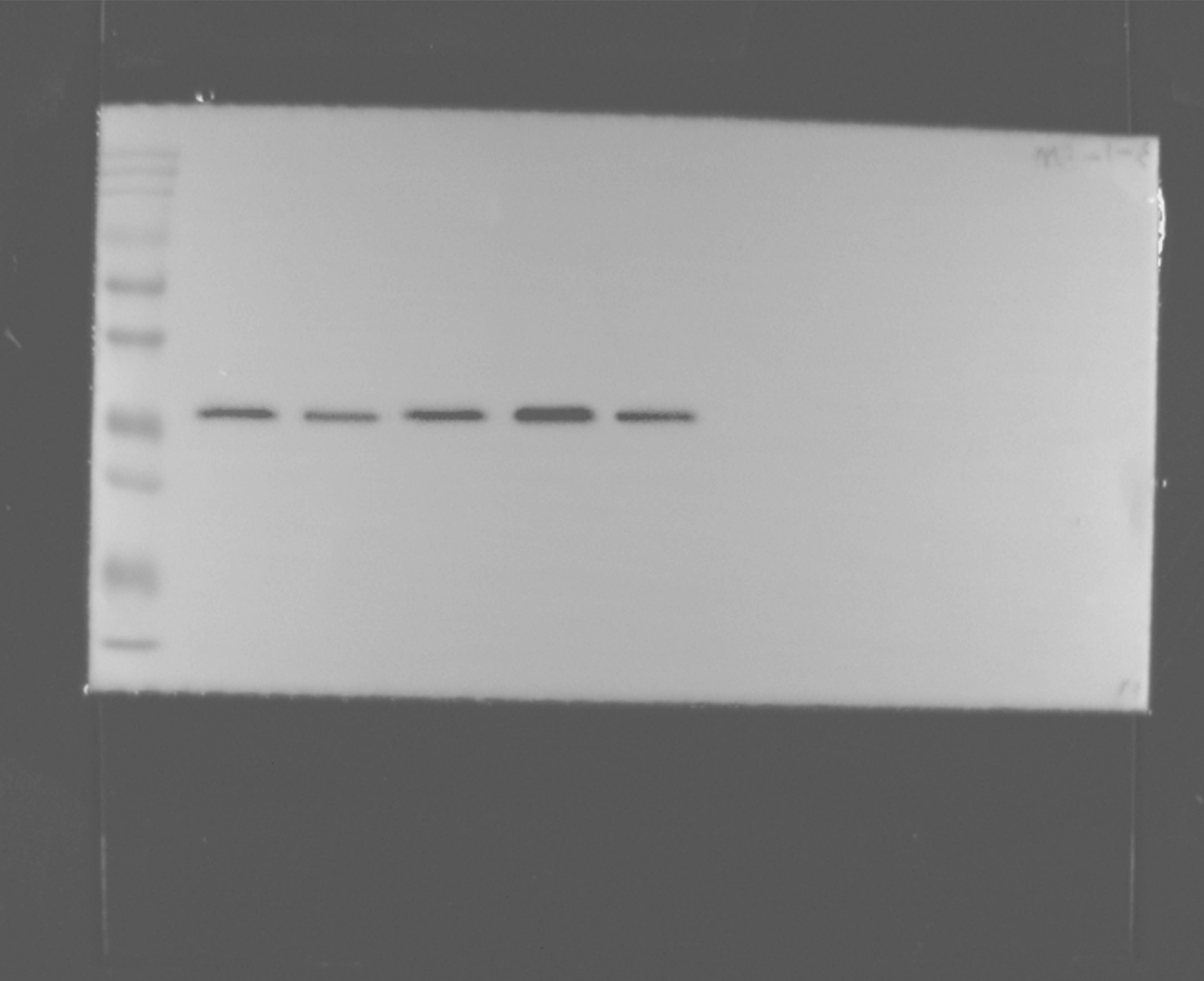

Supplement: Supplementary file 1 — Supplementary Material 1. [file 12876_2025_3836_MOESM1_ESM.zip › full uncropped Gels and Blots image/GSDMD-NT 31KDa -1.tif]

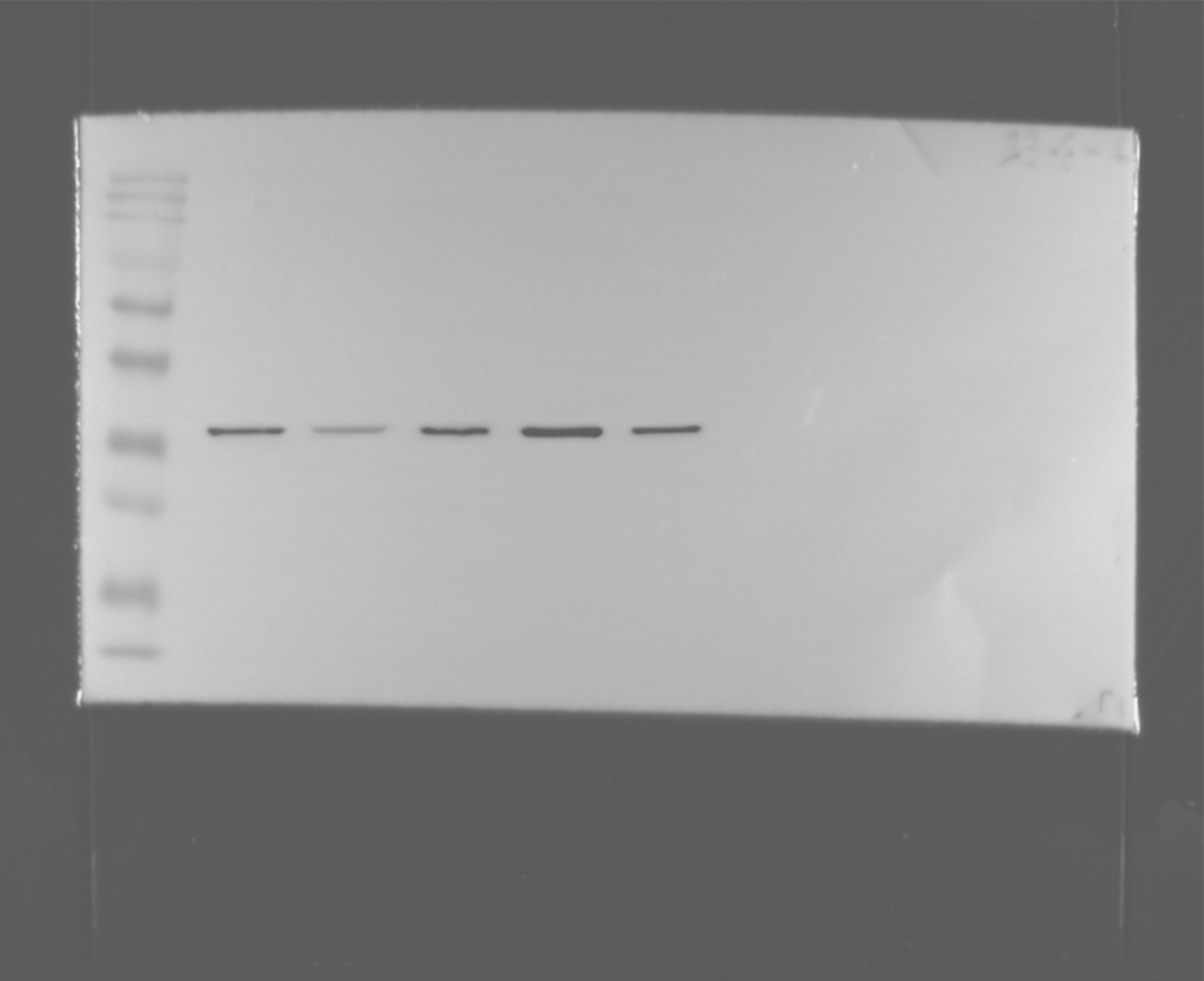

Supplement: Supplementary file 1 — Supplementary Material 1. [file 12876_2025_3836_MOESM1_ESM.zip › full uncropped Gels and Blots image/GSDMD-NT 31KDa -2.tif]

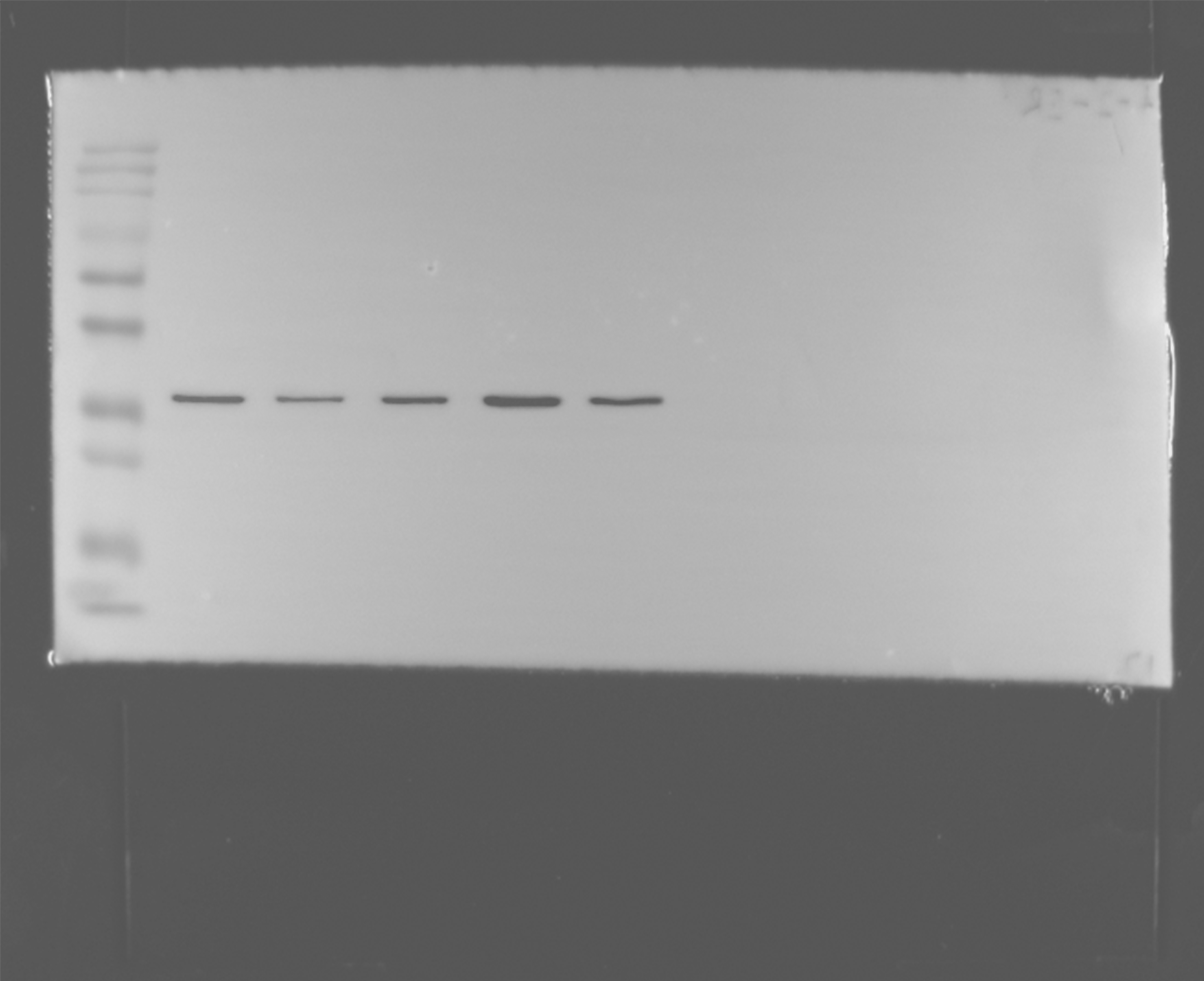

Supplement: Supplementary file 1 — Supplementary Material 1. [file 12876_2025_3836_MOESM1_ESM.zip › full uncropped Gels and Blots image/GSDMD-NT 31KDa -3.tif]

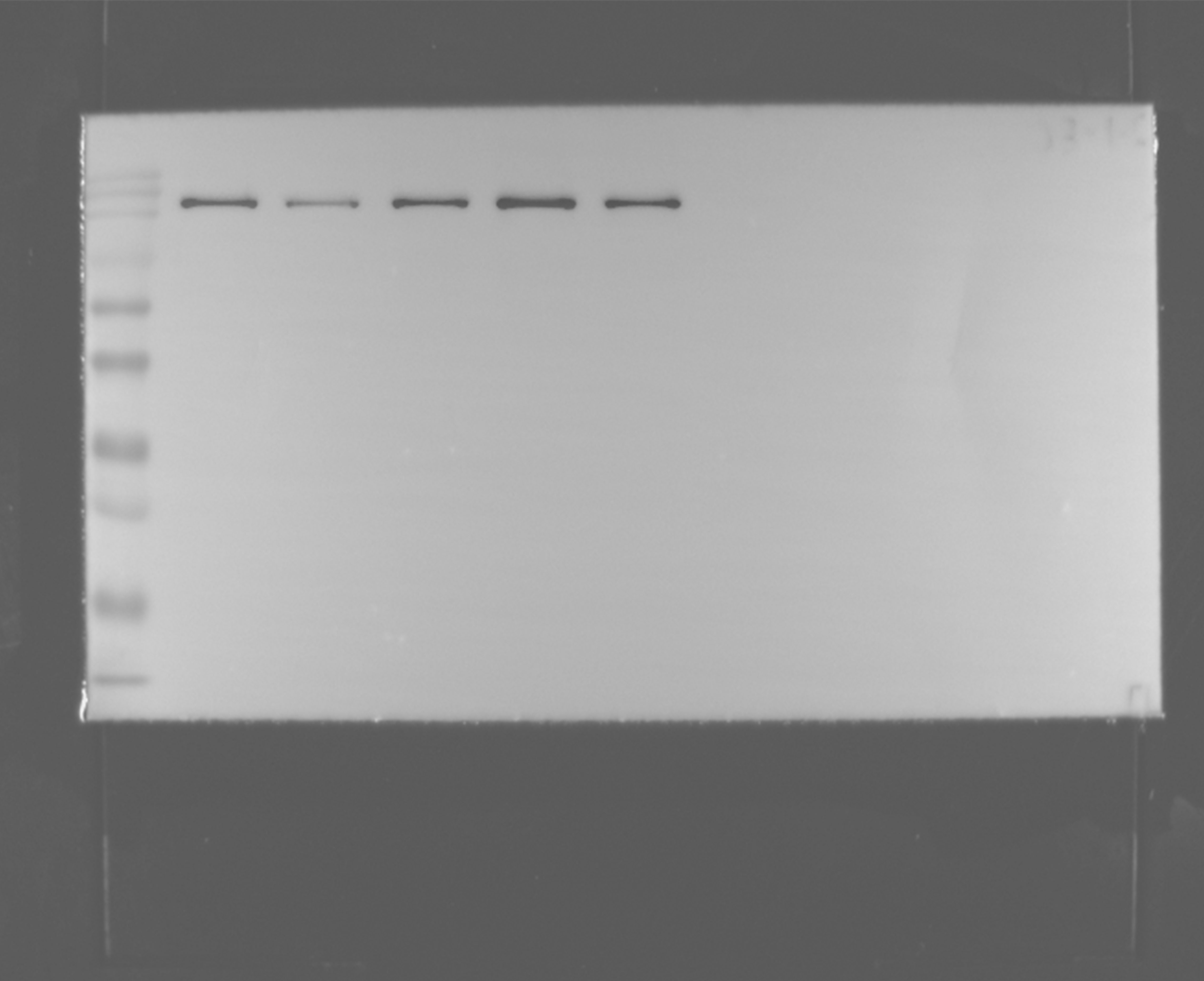

Supplement: Supplementary file 1 — Supplementary Material 1. [file 12876_2025_3836_MOESM1_ESM.zip › full uncropped Gels and Blots image/NLRP3 118KDa -1.tif]

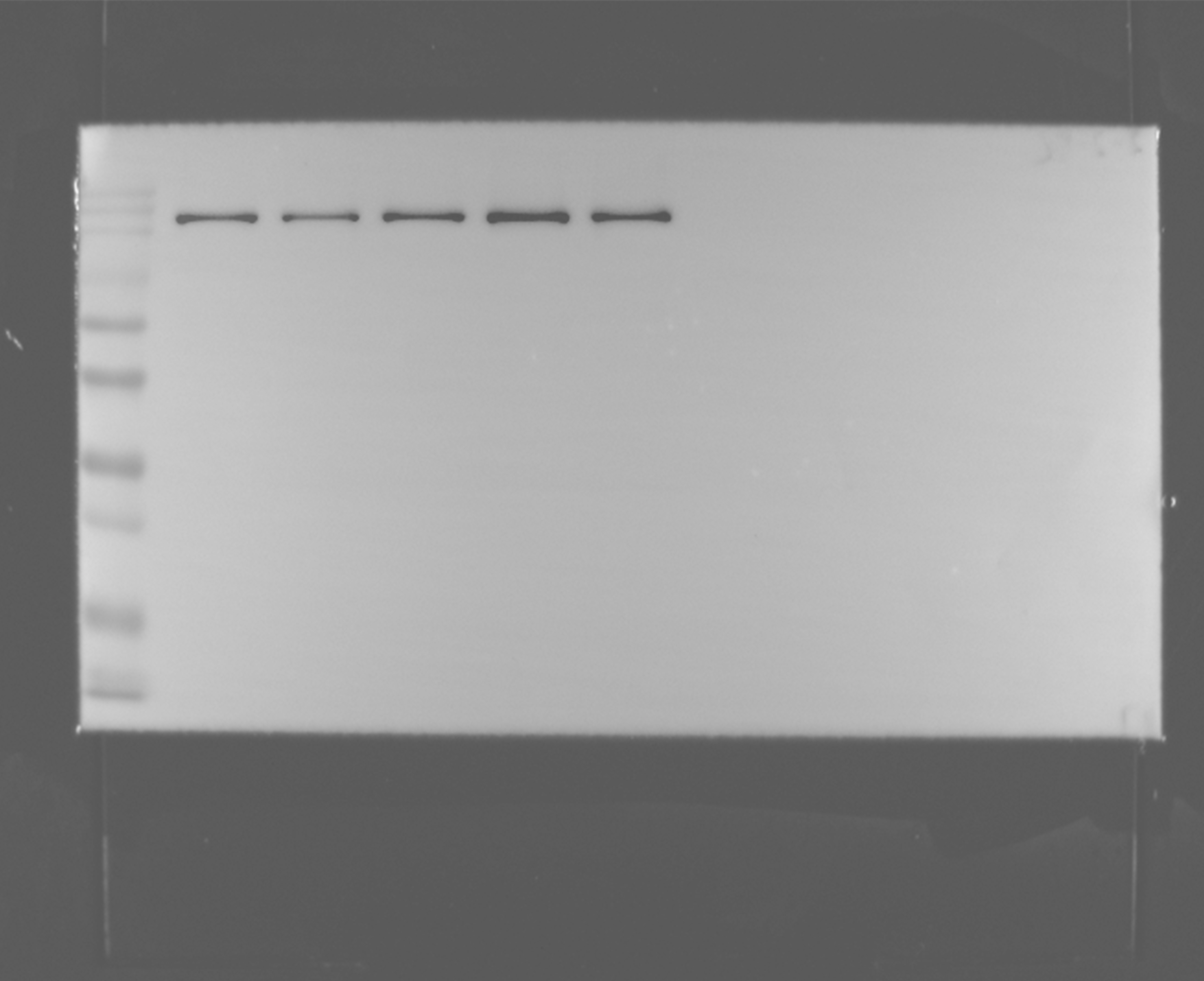

Supplement: Supplementary file 1 — Supplementary Material 1. [file 12876_2025_3836_MOESM1_ESM.zip › full uncropped Gels and Blots image/NLRP3 118KDa -2.tif]

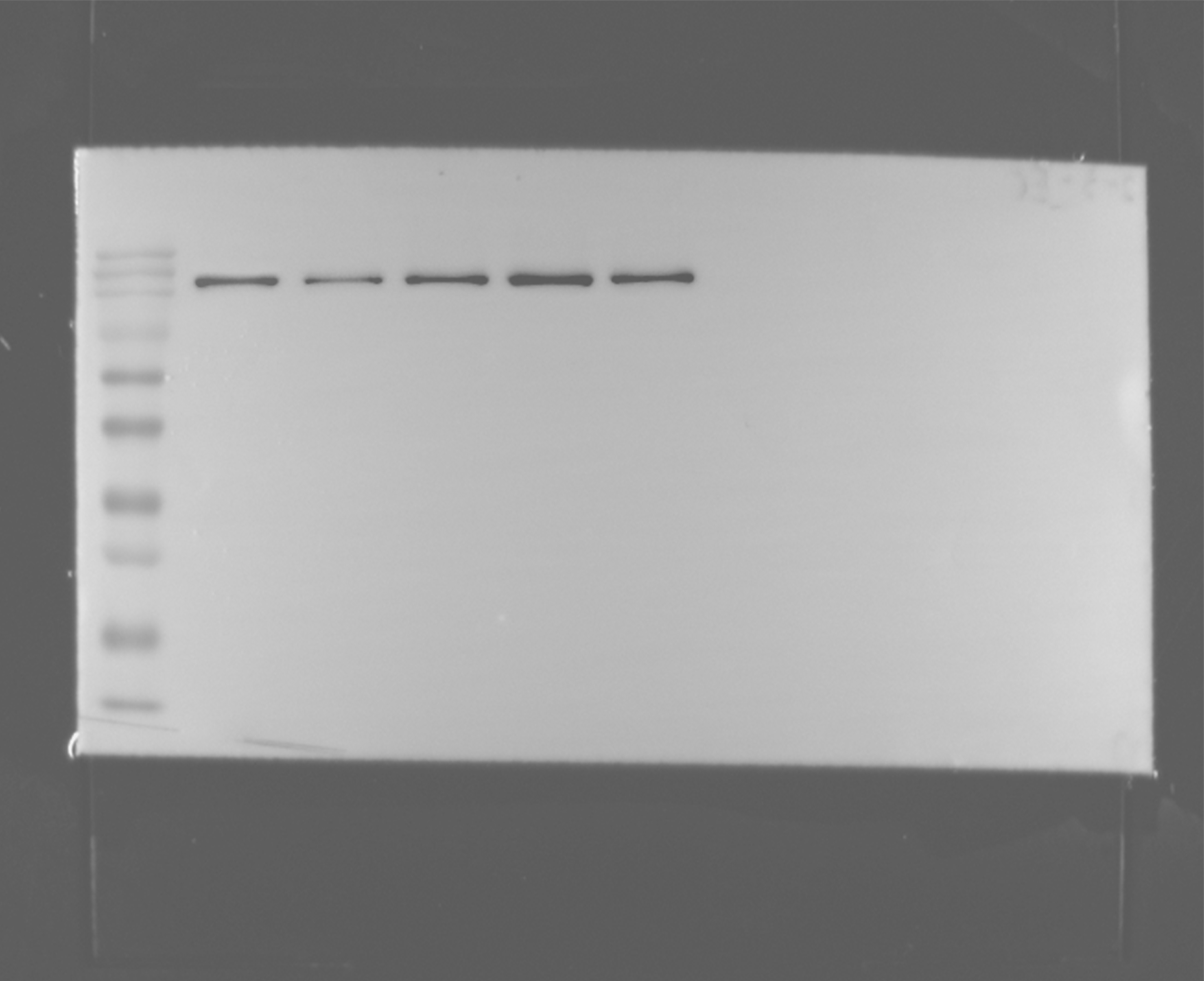

Supplement: Supplementary file 1 — Supplementary Material 1. [file 12876_2025_3836_MOESM1_ESM.zip › full uncropped Gels and Blots image/NLRP3 118KDa -3.tif]

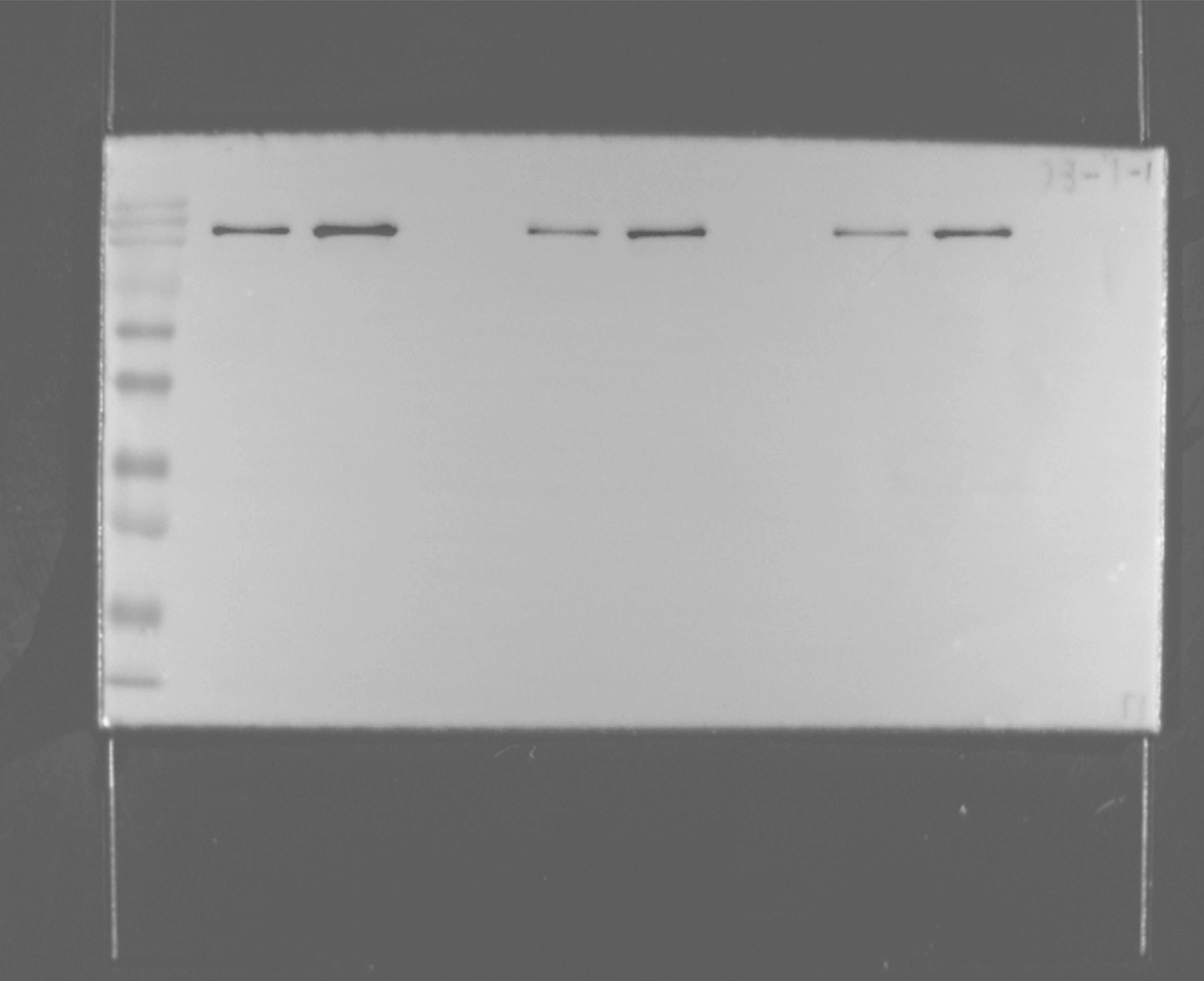

Supplement: Supplementary file 1 — Supplementary Material 1. [file 12876_2025_3836_MOESM1_ESM.zip › full uncropped Gels and Blots image/NLRP3 118KDa 01-03.tif]

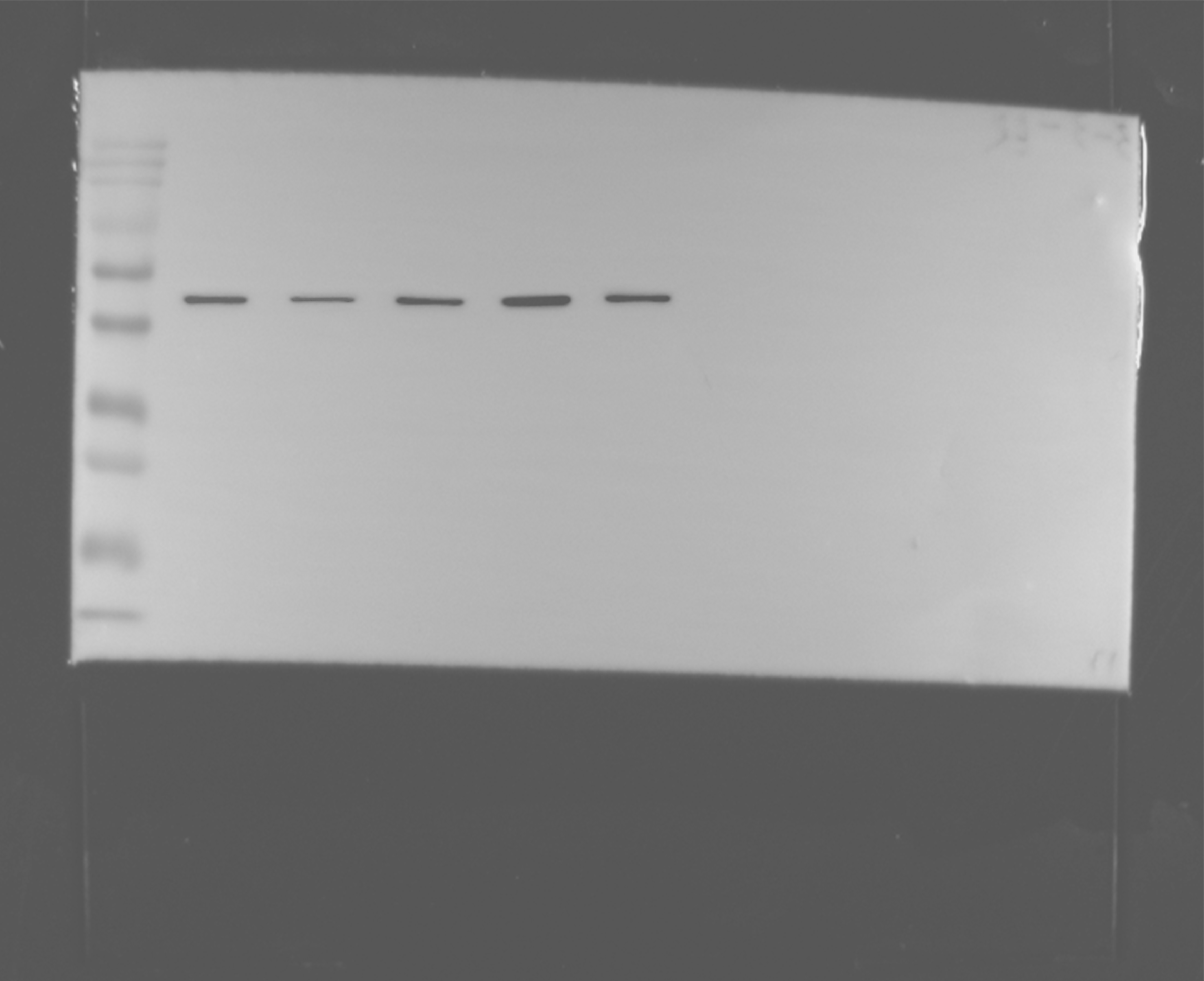

Supplement: Supplementary file 1 — Supplementary Material 1. [file 12876_2025_3836_MOESM1_ESM.zip › full uncropped Gels and Blots image/caspase-1 45KDa -1.tif]

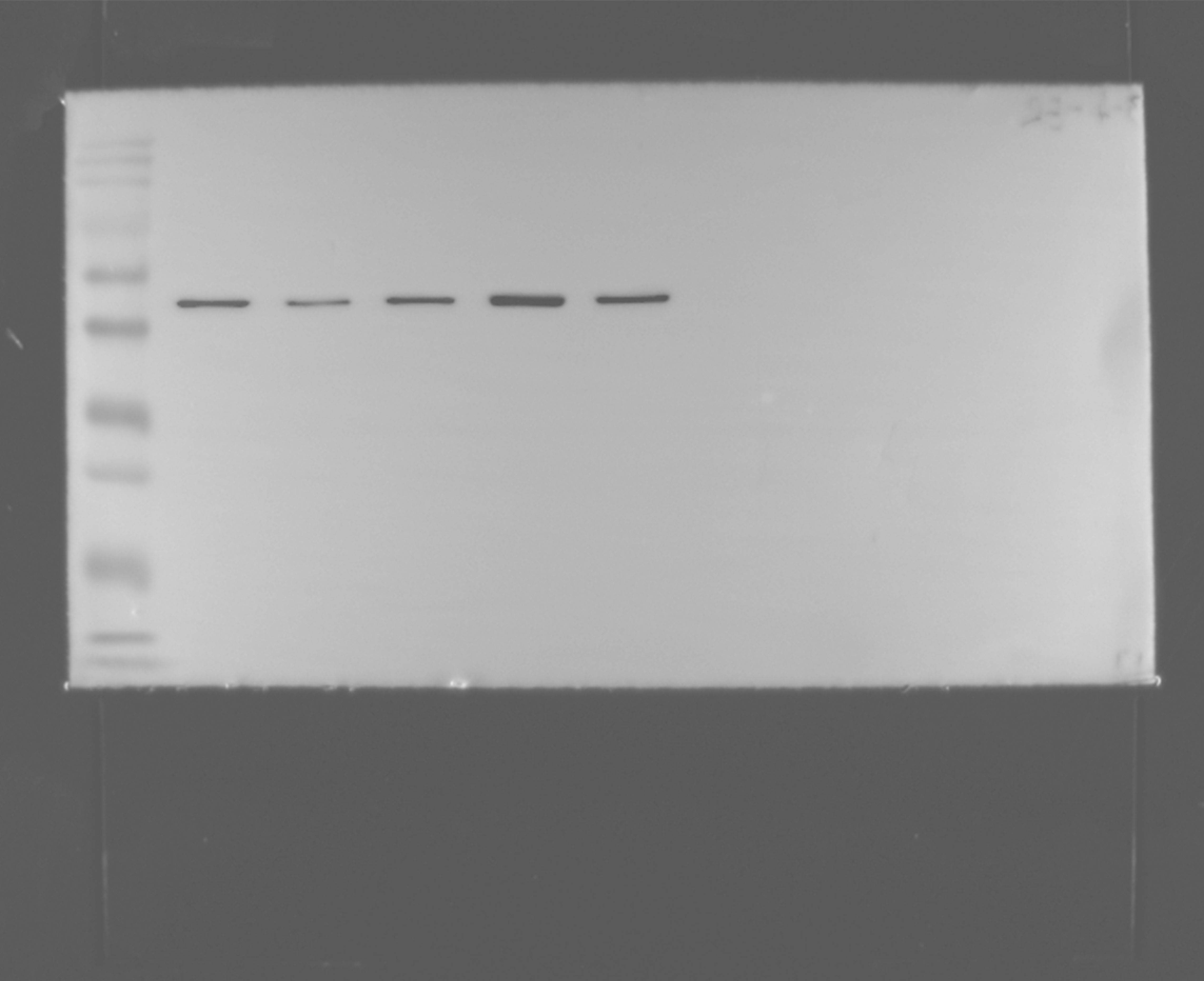

Supplement: Supplementary file 1 — Supplementary Material 1. [file 12876_2025_3836_MOESM1_ESM.zip › full uncropped Gels and Blots image/caspase-1 45KDa -2.tif]

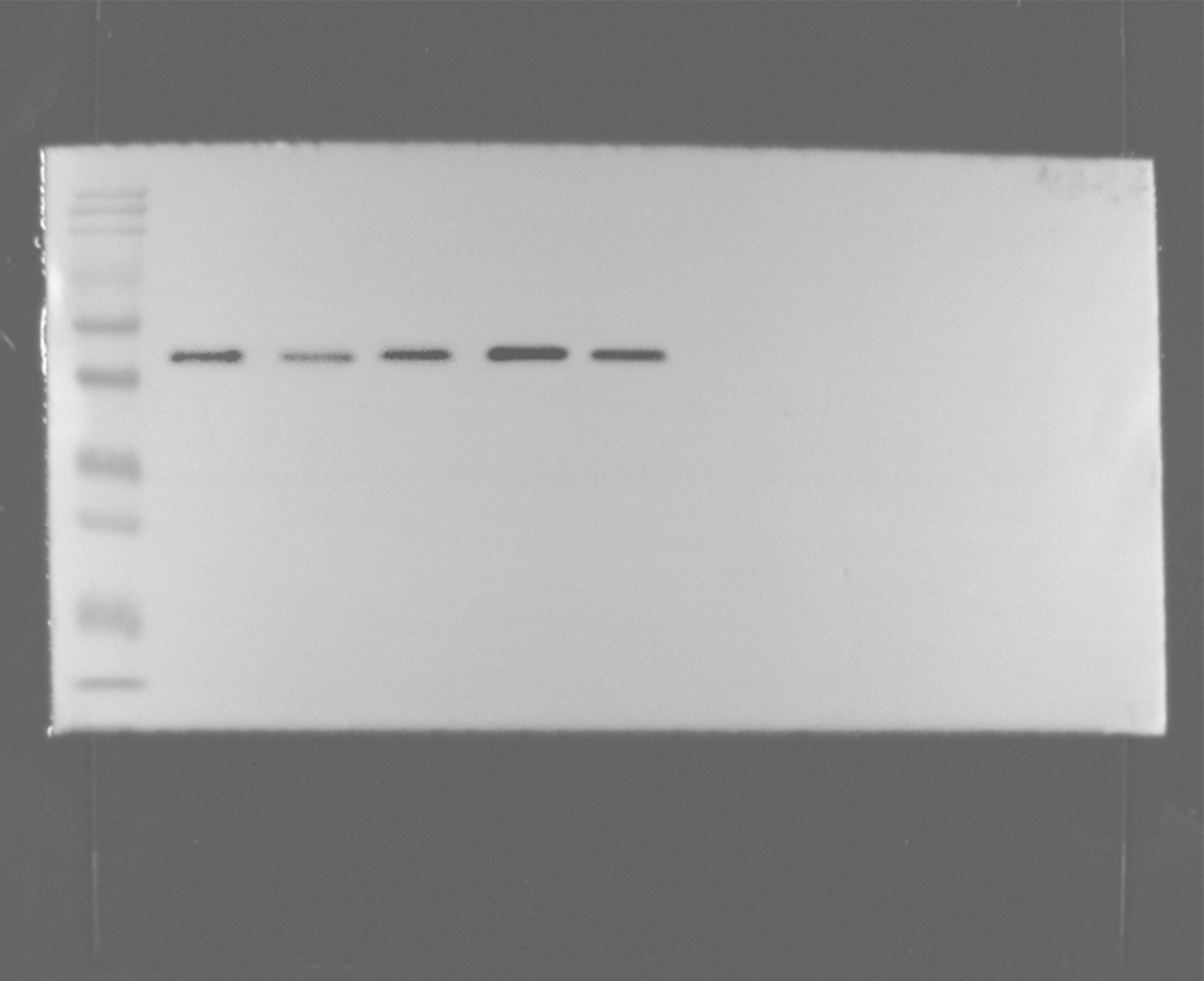

Supplement: Supplementary file 1 — Supplementary Material 1. [file 12876_2025_3836_MOESM1_ESM.zip › full uncropped Gels and Blots image/caspase-1 45KDa -3.tif]

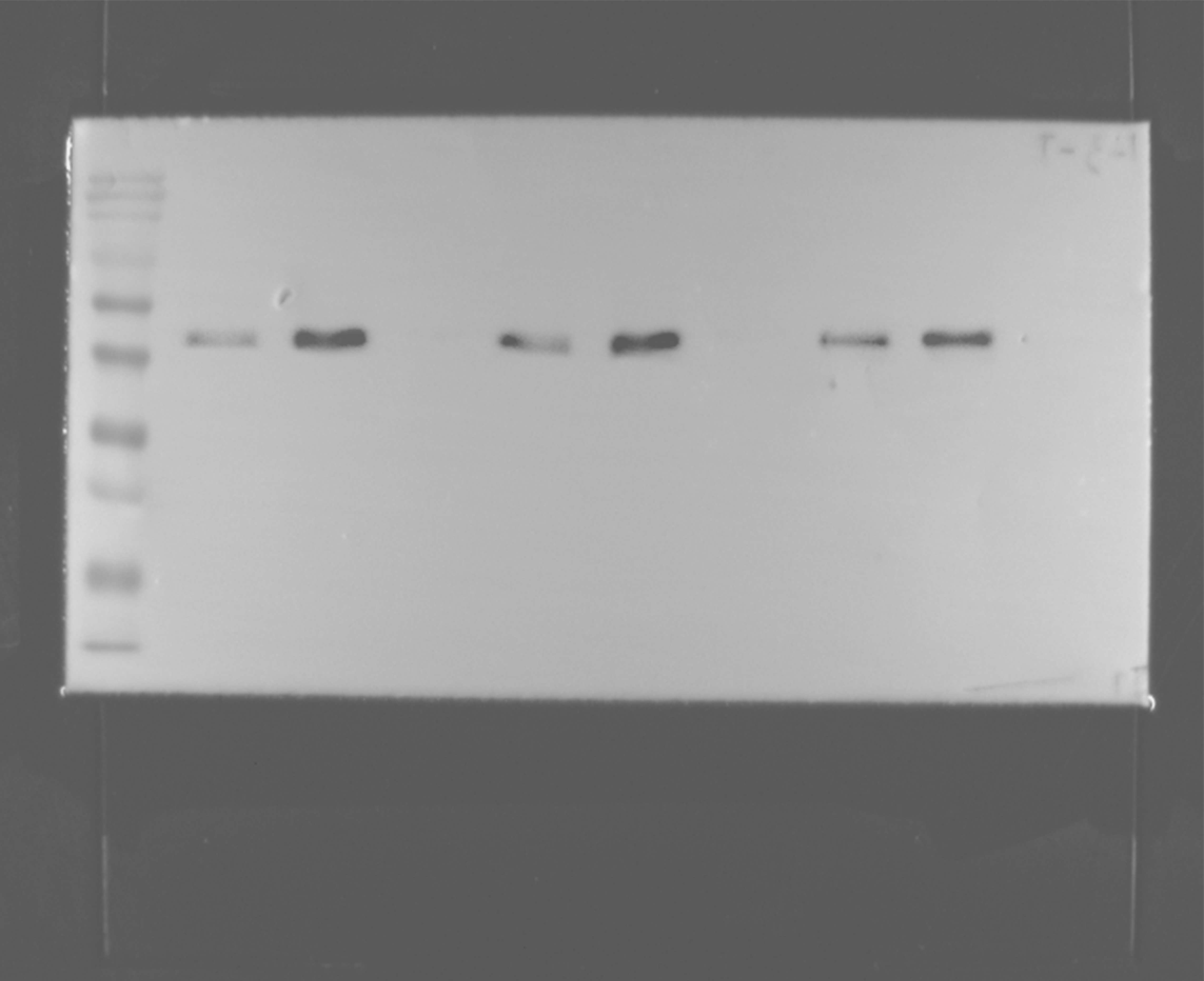

Supplement: Supplementary file 1 — Supplementary Material 1. [file 12876_2025_3836_MOESM1_ESM.zip › full uncropped Gels and Blots image/caspase-1 45KDa 01-03.tif]

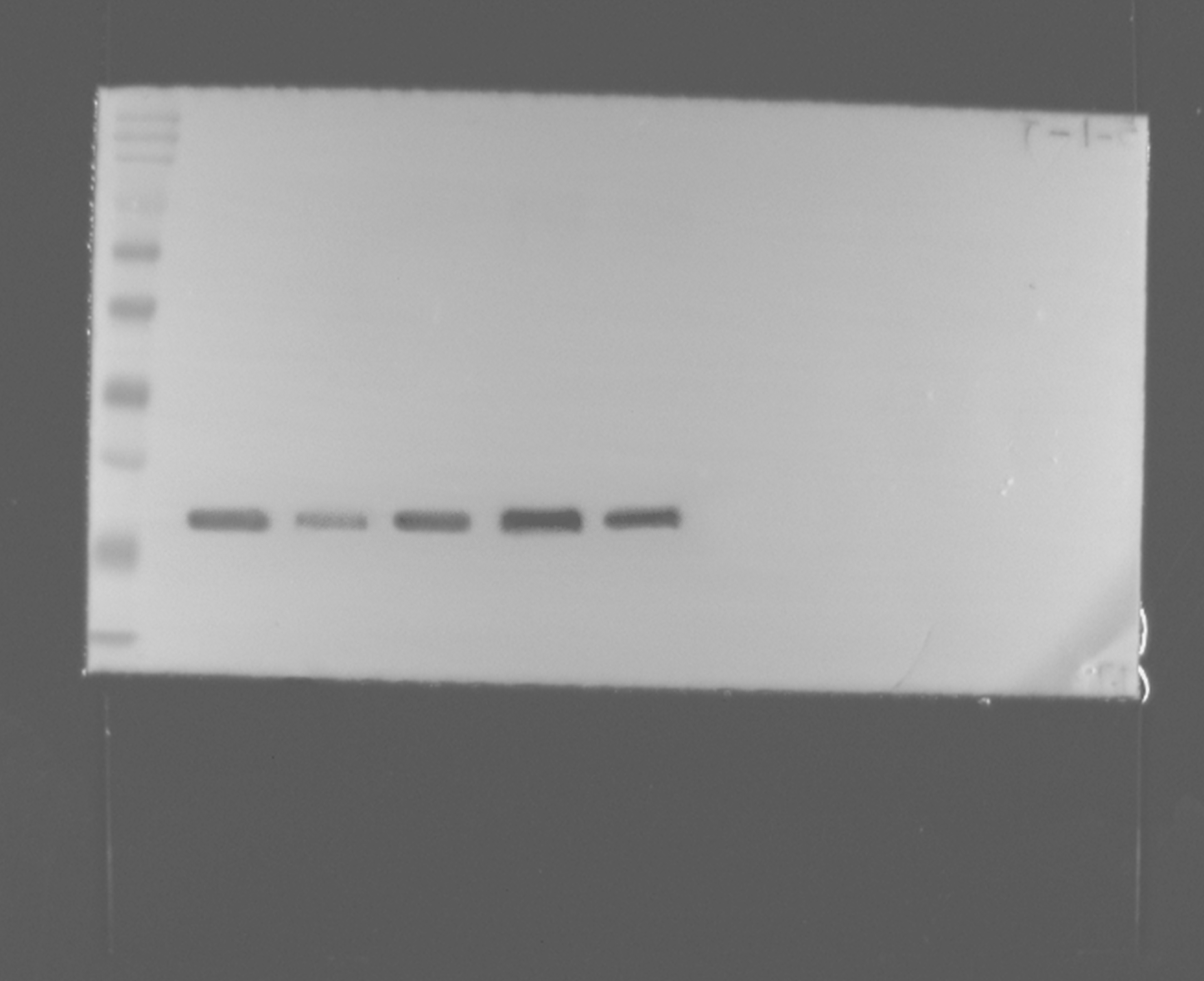

Supplement: Supplementary file 1 — Supplementary Material 1. [file 12876_2025_3836_MOESM1_ESM.zip › full uncropped Gels and Blots image/cleaved caspase-1 20KDa -1.tif]

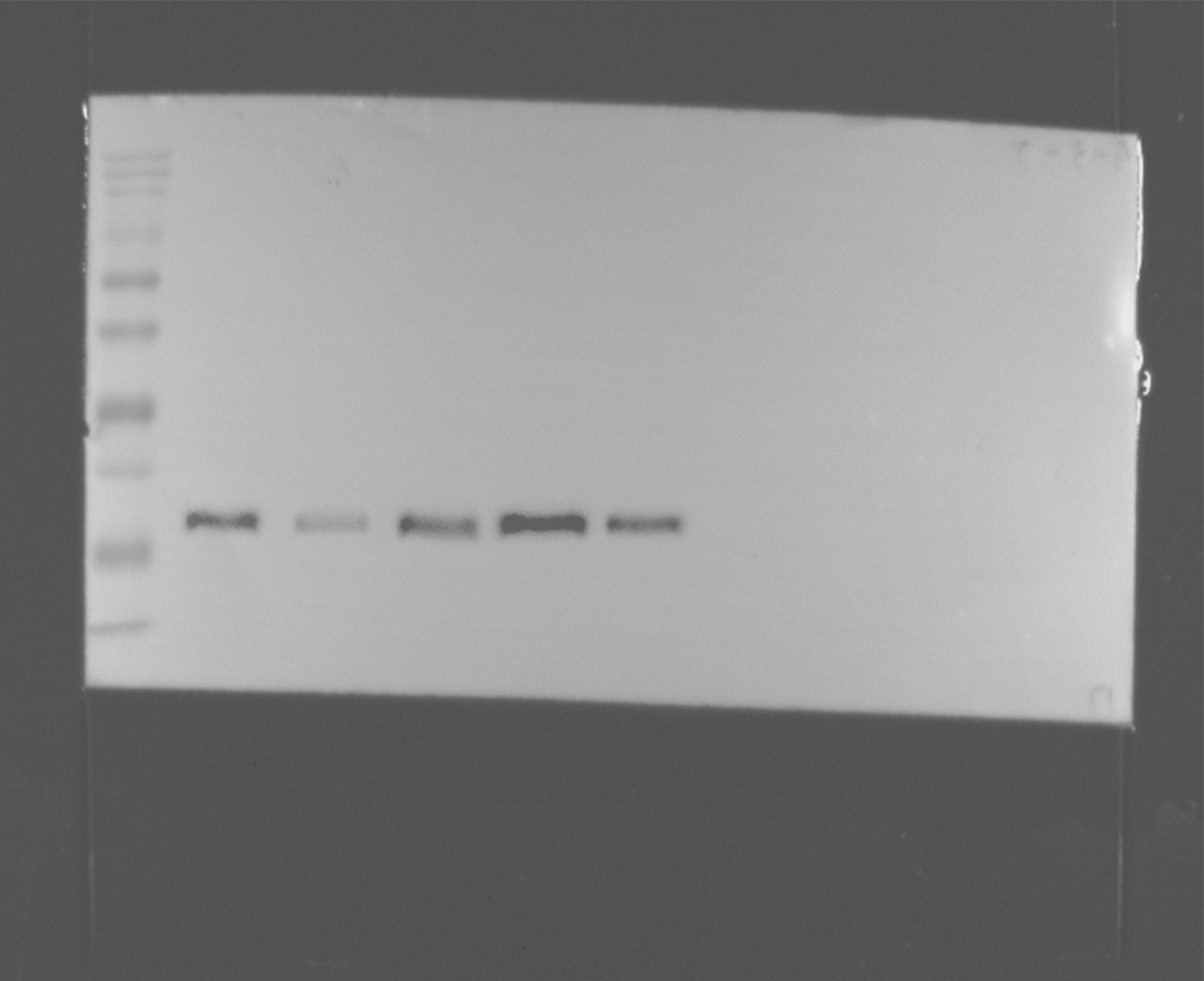

Supplement: Supplementary file 1 — Supplementary Material 1. [file 12876_2025_3836_MOESM1_ESM.zip › full uncropped Gels and Blots image/cleaved caspase-1 20KDa -2.tif]

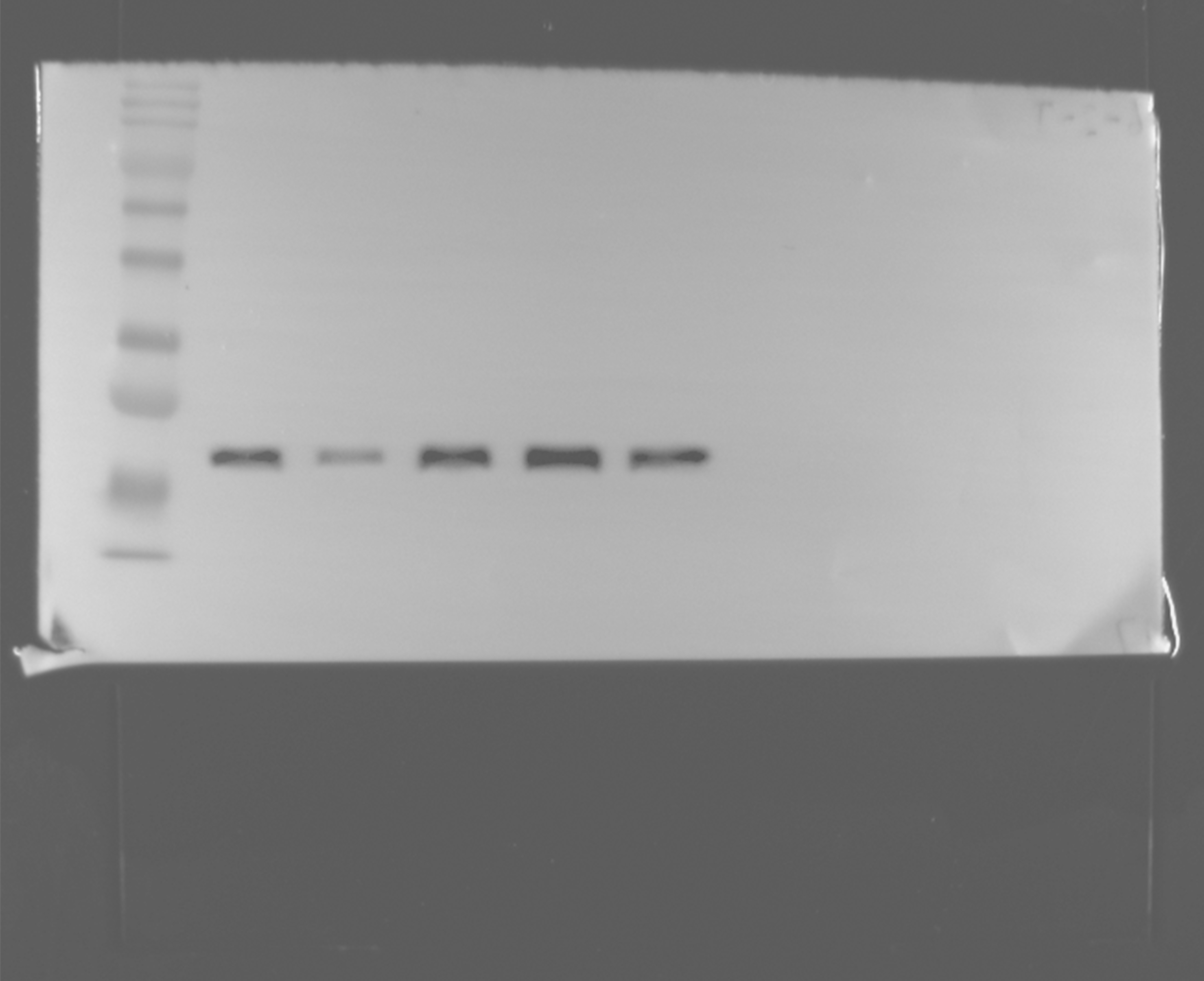

Supplement: Supplementary file 1 — Supplementary Material 1. [file 12876_2025_3836_MOESM1_ESM.zip › full uncropped Gels and Blots image/cleaved caspase-1 20KDa -3.tif]

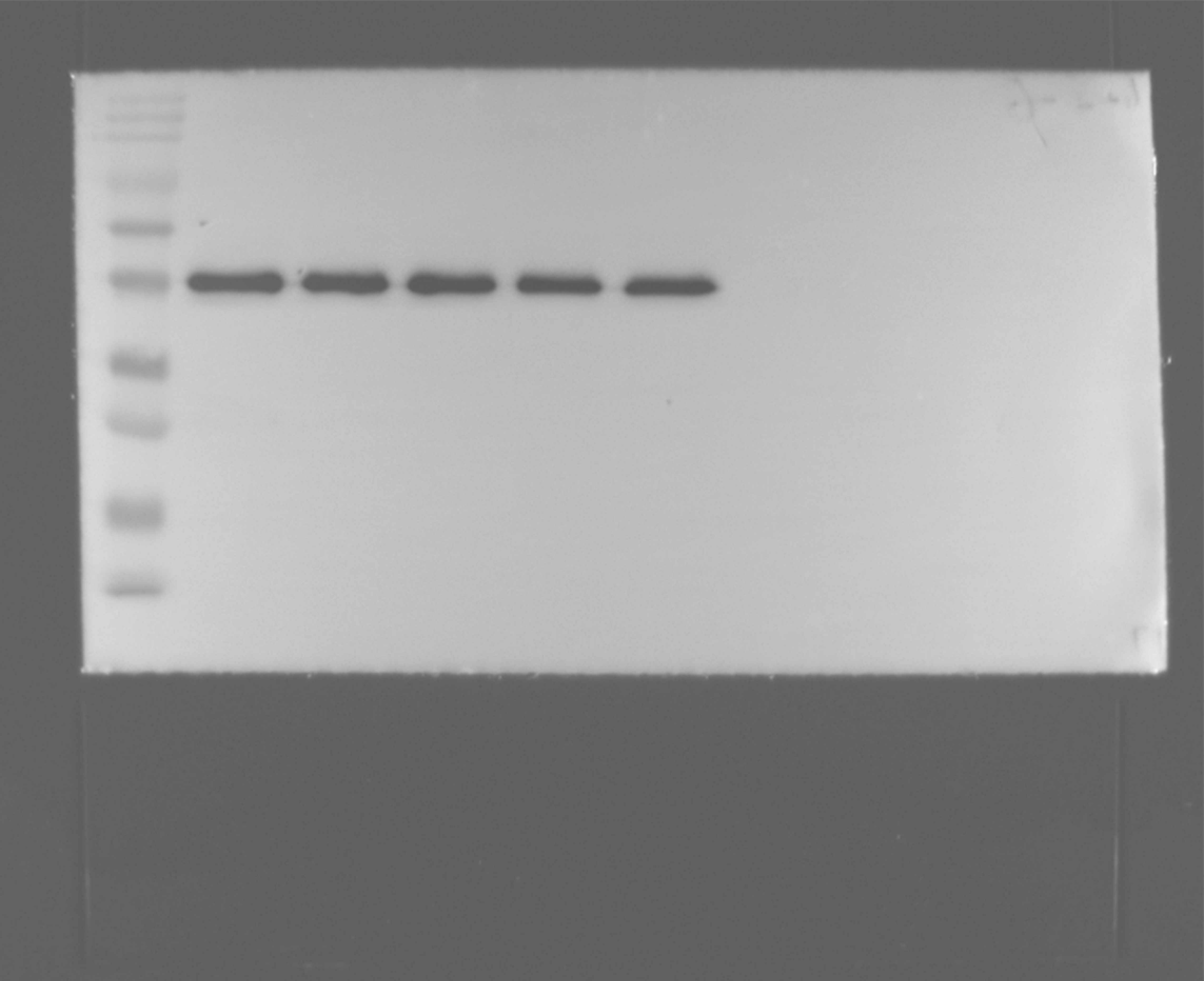

Supplement: Supplementary file 1 — Supplementary Material 1. [file 12876_2025_3836_MOESM1_ESM.zip › full uncropped Gels and Blots image/β-actin 42KDa -1.tif]

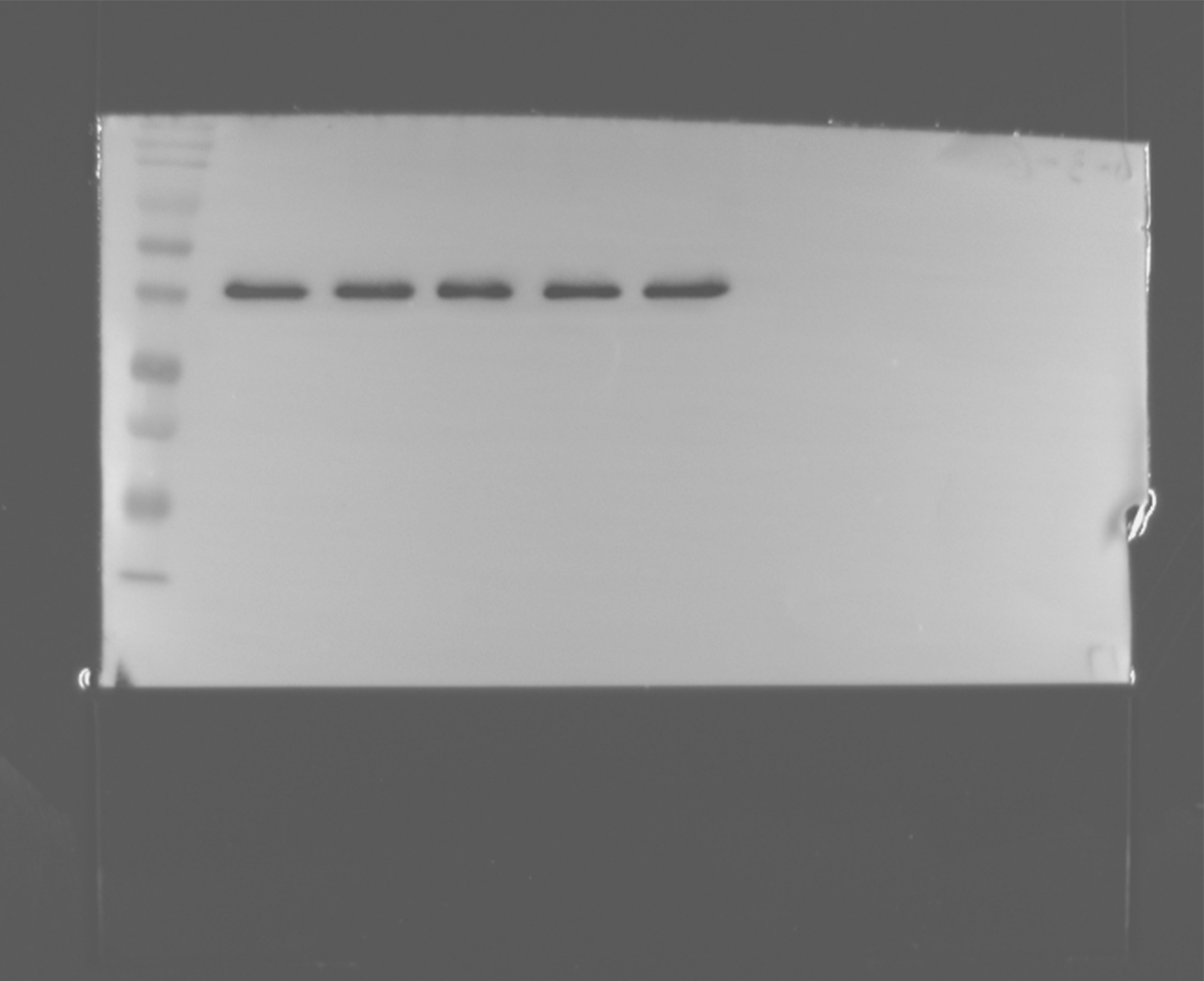

Supplement: Supplementary file 1 — Supplementary Material 1. [file 12876_2025_3836_MOESM1_ESM.zip › full uncropped Gels and Blots image/β-actin 42KDa -2.tif]

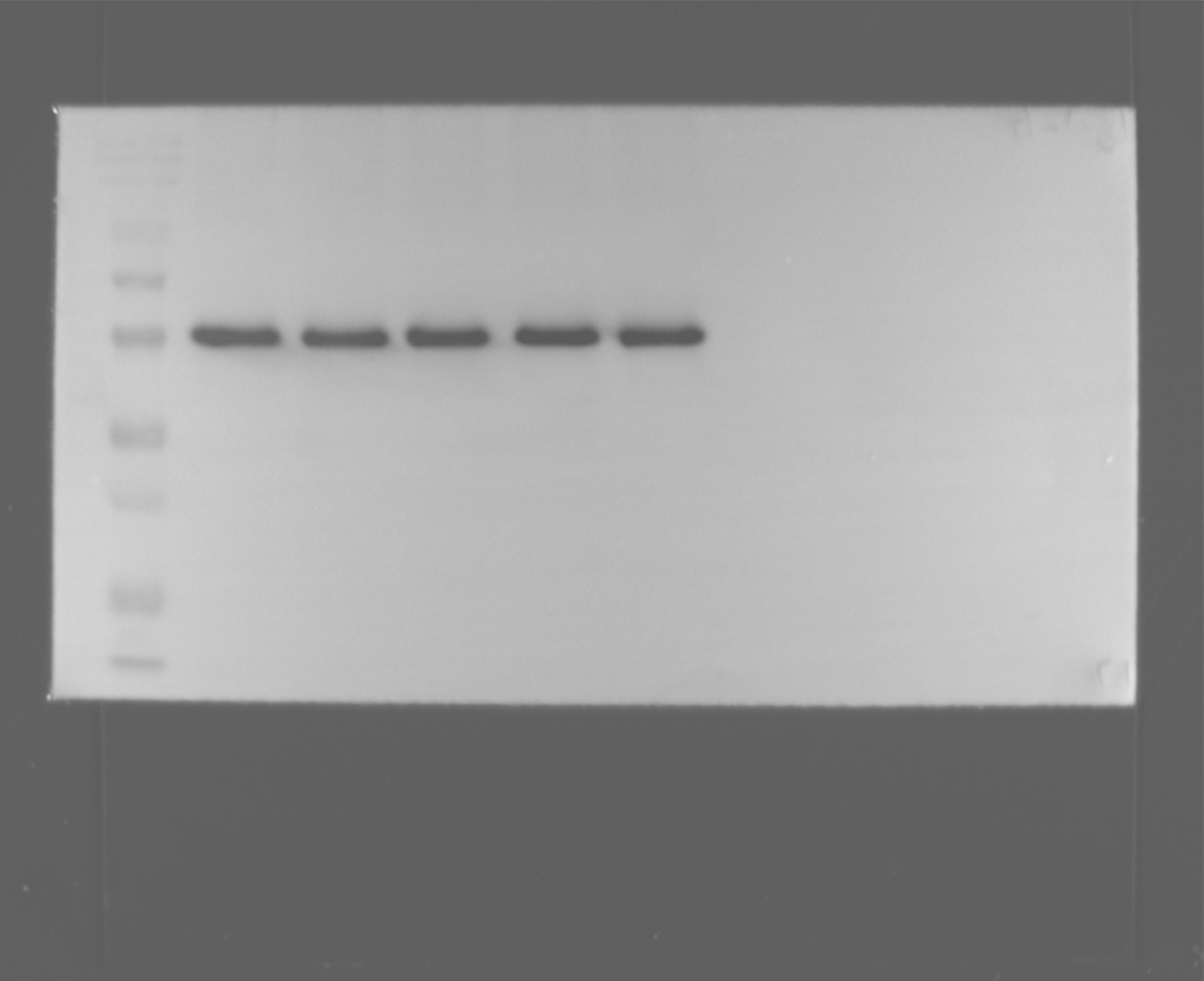

Supplement: Supplementary file 1 — Supplementary Material 1. [file 12876_2025_3836_MOESM1_ESM.zip › full uncropped Gels and Blots image/β-actin 42KDa -3.tif]

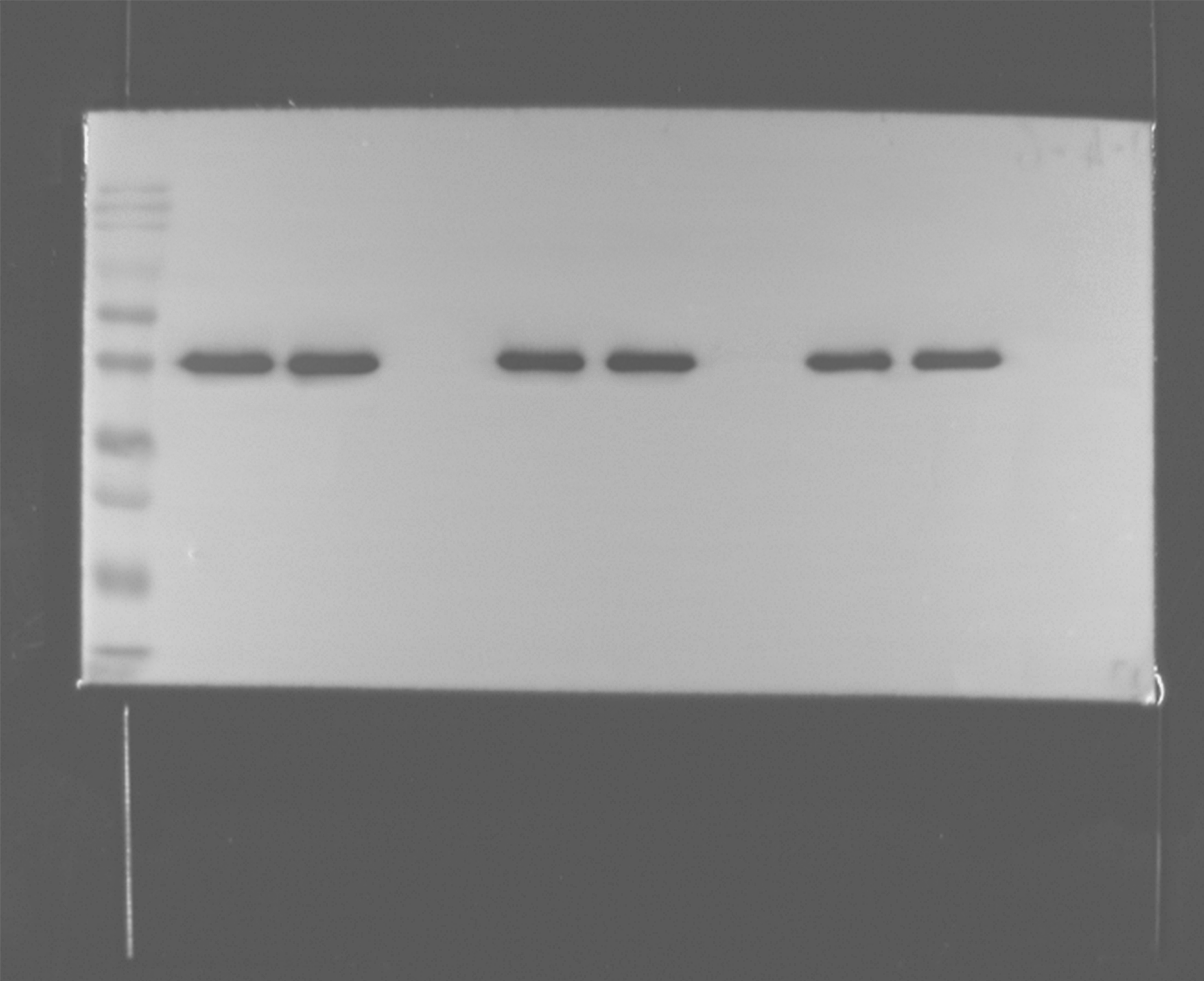

Supplement: Supplementary file 1 — Supplementary Material 1. [file 12876_2025_3836_MOESM1_ESM.zip › full uncropped Gels and Blots image/β-actin 42KDa 01-3.tif]
